# The Use of Behavioral Reconsolidation Interference in Depressive Disorders. A Double‐Blinded Randomized Controlled Experimental Registered Report

**DOI:** 10.1111/psyp.70217

**Published:** 2026-01-28

**Authors:** André Forster, Johannes Rodrigues, Billy Sperlich, Johannes Hewig

**Affiliations:** ^1^ Department of Differential Psychology, Personality Psychology, and Psychological Diagnostics Institute of Psychology, Julius‐Maximilians University of Wuerzburg Würzburg Bavaria Germany; ^2^ Department of Integrative & Experimental Exercise Science & Training Institute of Sport Sciences, University of Wuerzburg Würzburg Bavaria Germany

## Abstract

Depressive disorders often show recurrent courses that cannot be sufficiently prevented by existing therapeutic protocols. In other affective disorders, recurrence has been linked to three mechanisms –spontaneous recovery, accelerated new/relearning, and reinstatement– which are related to the preservation of disorder‐related memory traces even through successful extinction‐based interventions. Reconsolidation‐interference protocols aim to directly alter such traces by reactivating and destabilizing them before intervention. While this approach has shown benefits in fear, craving, and trauma‐related symptoms, its application to depression remains untested. To our knowledge, this study provides the first experimental evidence of its utility in depression‐like states. Sixty participants took part in a three‐day, three‐group, double‐blind randomized controlled trial. On day one, helplessness was induced using a modified unsolvable anagram task. On day two, participants were randomized into three groups undergoing different interventions while completing another cognitive demanding task: (1) extinction, where participants experienced success from start to finish; (2) reconsolidation, where participants briefly reexperienced failure before succeeding; or (3) reactivation, where failure repeated. On day three, the helplessness task was presented again to evaluate susceptibility for recurrence across conditions. Behavioral, self‐report, and EEG data were collected. Across test days, participants showed reduced motivation and performance, attributing failure to personal ability, confirming successful helplessness induction. However, interventions at day two produced no robust group differences on behavioral, self‐report, or EEG measures. Exploratory analyses suggested that brain‐derived neurotrophic factor (BDNF) levels may have mediated outcomes. Findings do not confirm reconsolidation‐based behavioral interference as effective for depression‐like helplessness. Nonetheless, exploratory results highlight BDNF as a potential mediator, warranting further study on its role in postretrieval extinction effects in depression.

## Impact Statement

Recurrence rates in depressive disorders remain high even after successful therapy. This study proposes an experimental protocol that assesses the possibility of targeted memory reconsolidation to modify long‐lasting vulnerability. The findings of this study provide first evidence concerning the potential and difficulties regarding applicability of this approach in the context of depression thereby paving the way for the translation of basic scientific results toward clinical application.

## 1 Introduction

Major Depression (MD) is one of the leading causes of health‐related burden worldwide (e.g., Ferrari et al. 2013; Santomauro et al. 2021; Liu et al. 2020). One reason for this is its highly recurrent course, in which subsequent episodes become increasingly probable with each endured episode (Angst and Preisig 1995; Angst et al. 1973; Kessing and Andersen 1999; Ehnvall and Agren 2002; Kessing et al. 2004). Unfortunately, although existing therapies can influence the course in the short term, detrimental long‐term outcomes are still likely, even after successful treatment with established therapeutic protocols (Keller and Shapiro 1981; Mueller et al. 1999; Hardeveld et al. 2010; Wojnarowski et al. 2019).

A promising new approach to address recurrent courses of affective disorders involves behavioral and chemical interference in labilized, previously consolidated memory traces that convey psychopathology (e.g., Kredlow et al. 2016; Pigeon et al. 2022). In sum, these *reconsolidation*‐based protocols are designed to introduce new information into established memory traces prior to a reconsolidation phase. While several studies were able to investigate the potency of this approach in fear‐ and anxiety‐related disorders (Agren et al. 2012; Soeter and Kindt 2012, 2015), posttraumatic stress disorder (Brunet et al. 2018; Gray et al. 2019; Roullet et al. 2021) or drug craving (Zhao et al. 2011; Becker et al. 2020; Das et al. 2015, 2019, 2018), to our knowledge, no study has been able to directly translate the corresponding protocols to the treatment of MD. This phenomenon may be attributed to two primary issues within this field of research:

First, the field of reconsolidation‐related research suffers from a general lack of reproducibility, reliability, and publication bias (e.g., Beckers and Kindt 2017; Lee et al. 2017; Schroyens et al. 2022). These issues have been shown not only in classical conditioning studies that were designed to emulate psychopathology (e.g., Bos et al. 2014; Chalkia et al. 2020; Elsey and Kindt 2021; Luyten and Beckers 2017), but also in learning tasks that may especially be potent in operationalizing retrieval‐dependent reconsolidation effects (e.g., Hardwicke et al. 2016). As a result, several reviews and meta‐analyses have focused on methodological issues and boundary conditions of reconsolidation effects that may explain these mixed results (Auber et al. 2013; Dunbar and Taylor 2017; Lee et al. 2017; Schroyens et al. 2022). Second, in the field of MD research, an additional set of methodological issues may arise from the heterogeneous nature of the disorder. Along this line, differences in the etiology or phenomenology of symptoms across patients may introduce even greater difficulties in terms of replicable results (e.g., Fried 2017).

Therefore, to improve reproducibility and transparency, additionally to this registered report's main manuscript presented here, an extensive supplemental discussion of each methodological choice is provided at: https://doi.org/10.17605/OSF.IO/NT65F.

### 1.1 Classical Conditioning as a Model of Pathology

Contemporary models of psychopathology frequently adopt a conditioning framework, viewing psychiatric disorders like fear and anxiety disorders as the outcome of repeated pairings between a conditioned stimulus (CS) and an unconditioned stimulus (US). Within this perspective, such pairings create an associative memory trace that is progressively strengthened with each occurrence, gradually intensifying behavioral and physiological responses to the CS (see, e.g., Beckers et al. 2013). Accordingly, symptoms of fear and anxiety, including withdrawal or panic, have long been conceptualized as emerging from classically conditioned CS‐US associations, which are subsequently reinforced through operant mechanisms (e.g., avoidance of the CS, thereby preventing disconfirmation of expectations; Fullana et al. 2020; Mowrer 1956). Similarly, depressive responses such as rumination and self‐deprecation may become linked to co‐occurring experiences like negative mood (Watkins and Nolen‐Hoeksema 2014) or ambiguous cues (e.g., Moser et al. 2012), potentially establishing a conditioned vulnerability factor for MD.

In extinction‐based therapeutic protocols, the CS is repeatedly presented in the absence of the US, thereby challenging patients to test the validity of their previously learned CS‐US associations. Through this process, existing associations may weaken while new, more adaptive ones may emerge (e.g., Quirk and Mueller 2008). For instance, an individual who has learned to associate social interaction with rejection may habitually avoid social contexts (conditioned response). Over the course of therapy, such avoidance typically decreases as patients develop alternative coping strategies and social skills, which in turn reduce the likelihood of negative social feedback, even when these situations are not avoided. The following encounters that potentially disconfirm prior expectations generate prediction errors (i.e., instances of surprise), which can then be leveraged to update maladaptive beliefs. This mechanism has been shown to support the formation of new associations concerning the reduced or absent predictive value of the CS (in this example, social interaction) for the occurrence of the US, such as rejection (e.g., Hermans et al. 2006).

### 1.2 Preventing Reinstatement, Accelerated Relearning and Spontaneous Recovery

Importantly, a substantial body of evidence indicates that large prediction errors are more likely to generate *new* associations, encoded as distinct memory traces, rather than directly updating preexisting ones (Bouton and Moody 2004; Gershman et al. 2010; Fernández et al. 2016; Sevenster et al. 2014). Consequently, although extinction‐based exposure therapy for fear and anxiety disorders may temporarily alleviate symptoms by reinforcing the recently acquired, adaptive CS‐US association, the originally established, maladaptive CS‐US associations remain intact, though their influence may be attenuated. As a result, symptom‐related memory traces retain the potential to reemerge through mechanisms such as spontaneous recovery, rapid reacquisition, or reinstatement (e.g., following another episode of rejection), which may ultimately facilitate the recurrence of psychiatric symptoms (Rescorla 1988, 2001; Bouton and Moody 2004).

To address this challenge, reconsolidation‐based protocols aim to directly modify the original memory trace rather than merely inhibiting it through the establishment of competing new associations. This is typically achieved by briefly reactivating the memory through a reminder cue (e.g., by presenting the original CS‐US pairing once) before initiating extinction training. The reminder is thought to “activate” the existing association, after which the introduction of a prediction error during extinction training renders the memory trace amenable to updating (e.g., Auber et al. 2013). The temporal window during which a reactivated memory remains labile and open to the incorporation of new information or disruption is referred to as the *reconsolidation window* (RW; Pedreira et al. 2004; Vaverková et al. 2020; Walker et al. 2003). Within this window, previously consolidated information can be modified before it stabilizes again through reconsolidation. Extinction‐based interventions delivered during the RW are therefore theorized to alter the original vulnerability itself, rather than creating a competing memory trace, thereby offering a potentially more long‐lasting therapeutic effect (e.g., Soeter and Kindt 2015; Johnson and Casey 2015).

### 1.3 Sensitization

Reconsolidation refers to the process by which an already consolidated memory trace is returned to a labile state and subsequently stabilized once again. In this context, reexperiencing the original association (e.g., social interaction followed by rejection) can be viewed as a reconsolidation‐based updating process. Even when no new information is introduced prior to reconsolidation, such reminders may nevertheless alter the patient's memory trace by strengthening the original association through re‐reinforcement of prior conditioning (sensitizing the organism). This mechanism is exemplified by Keyan and Bryant (2017b), who demonstrated that experimentally induced “traumatic” memories could be amplified through reconsolidation. Conceptually, this implies that each reactivation of a vulnerability‐related association increases the patient's risk for future depressive episodes, as the association is repeatedly reinforced and reconsolidated. Over time, such vulnerabilities become more prone to accelerated relearning, spontaneous recovery, or reinstatement. Consistent with this view, a substantial body of evidence supports the hypothesis that recurrent episodes contribute to progressive sensitization, as outlined in Robert Post's kindling/sensitization theory (e.g., Post 1985, 2007a, 2007b, 2010). To prevent such sensitization, therapeutic protocols thus need to focus not only on labilizing existing memory traces, but also on adding new information before allowing reconsolidation, as discussed in the previous section with regard to postretrieval extinction protocols.

## 2 Reconsolidation in Learned Helplessness as a Model of Depression^1^

To test the utility of reconsolidation‐based protocols in the context of depression, a paradigm that is similar to existing reconsolidation‐based protocols is the most promising option. Here, fear‐ and anxiety‐related research may be seen as a suitable prototype as its well‐described experimental learning protocols allow trial‐by‐trial measurement of conditioning and subsequent responses (e.g., Luyten and Beckers 2017). Courses of responses on a trial‐by‐trial level are especially relevant as the main distinction between successful extinction training within and outside of the RW should lead to differences in the (re)learning/(re)conditioning curves after the intervention. Consider the freezing response in rodents to a fear‐conditioned CS. During the initial conditioning phase, freezing should gradually increase as the CS‐US association is learned. During extinction training, the freezing response should then decrease significantly, as the CS is no longer predictive of the US. However, according to the findings discussed above, presentation of another CS‐US pairing after this successful extinction training should still invoke considerably greater freezing responses than were seen in the first CS‐US pairing during the initial conditioning phase, as the organism can now reactivate what was learned on that day, without having to go through the complete conditioning procedure once more. In other words, the original memory trace was preserved during extinction training stimulating reinstatement of the original fear even after successful intervention.

### 2.1 Learned Helplessness

A promising paradigm that links depressive symptoms to a similarly well‐defined trial‐by‐trial learning process is provided by the learned helplessness (LH) framework. According to this theory, the repeated perception of effort‐outcome non‐contingency leads to the establishment of a certain style of attribution (internal, global, and stable attribution of this incongruence), which in turn induces a reduction of effort, thereby entering a vicious cycle of overly passive behavior, negative thought, and affect (Maier and Seligman 2016). LH‐based paradigms were able to reproduce every DSM‐IV criterion of MD (except for suicidal ideation), highlighting the validity of the model (Maier and Seligman 2016).

A paradigm that has already been used to induce LH, which is also suitable for single‐trial analyses, is the unsolvable anagram task (Frankel and Snyder 1978; Snyder et al. 1981). Here, unsolvable anagrams, which are falsely declared to be solvable, are presented. As a result, through continuous failure, LH should eventually set in. In this regard, one criticism concerning the paradigm is that participants will not necessarily become helpless but simply stressed. Nonetheless, this stress should be associated with helplessness even though it may not take on its full dimensions. Despite this limitation, it can be assumed that unsolvable anagrams in principle lead to a helplessness‐like state that is characterized by negative affect, effortlessness, and passivity; a state that is conceptually different from fear responses.

### 2.2 General Methodological Considerations for the Present Study

The investigation of reconsolidation interference requires at least three test days, including an initial conditioning day, an intervention day, and a measurement occasion on a separate day that tests for effects of reinstatement, allowing for consolidation during sleep phases between assessments. Furthermore, comparison of at least three groups is required including one that undergoes a retrieval phase (reactivation of the helplessness) before being subject to a prediction error and an extinction protocol (reconsolidation group), another group that undergoes the same extinction protocol without a preceding retrieval phase (and prediction error; extinction‐control group) and one group that undergoes the procedure without an extinction phase after retrieval (reactivation control group). In sum, a three‐day (1. LH‐task, 2. intervention, 3. reinstatement test), three‐group (1. reconsolidation, 2. extinction, 3. reactivation) design emerges. An illustration of this concept can be found in Figure 2, depicting the final procedure of this study.

### 2.3 Unsolvable Anagram Task of the Present Study

To ensure interpretability of responses as helpless, they need to be maladaptive. Unfortunately, classical unsolvable anagram tasks may not be able to ensure this, as passivity and task disengagement may be viewed as adaptive responses to generally unsolvable tasks. One way to address this is the administration of occasionally solvable trials that allow for an analysis of the participants' effort to engage with the current trial despite having failed before. Hence, reaction time (RT) in these (solvable) trials can act as a continuously scaled indicator for passivity/helplessness in a controllable situation. Thus, in the experiment described below, all presented anagrams are per se solvable, while in most trials, the overall time limit is restricted to the point of unsolvability. However, without prior notice, some trials/anagrams had no time restriction imposed on them, allowing the interpretation of the RT (time needed to solve the anagram) as stated above.

### 2.4 Extinction Protocol

Following induction of helplessness beliefs (effort‐outcome non‐contingency), an extinction‐like protocol was used to stay as close to classical protocols in fear conditioning as possible. In the context of LH theory, experiencing controllability over an aversive event that would later be used to induce LH increases resilience against becoming helpless (Amat et al. 2010). Further, existing helpless responses of subjects may also be reversed by similar training procedures that are used in extinction learning, i.e., by presenting solvable tasks (“success therapy”; Nation and Massad 1978). Presenting demanding tasks with an opportunity to control their outcome thus seems to exhibit similar properties as CS‐no‐UCS pairing trials do in fear conditioning and extinction contexts. Therefore, in the following, in the context of LH, the *extinction protocol* refers to the presentation of solvable (but demanding) tasks.

### 2.5 Reconsolidation Induction

As stated above, a reminder (inducing retrieval), followed by a prediction error, is necessary to open an RW. Since the central conditioning experience in LH is the supposedly missing contingency between effort and positive results, once a helplessness belief was formed, presentation of a (seemingly) demanding task may successfully reactivate this conditioning experience. Unfortunately, this leads to the problem of designing an extinction‐based control group that does not accidentally also undergo reconsolidation based on retrieval of helplessness simply by *expecting* whatever task comes up to be overwhelmingly hard, which may then lead to an expectation error as they undergo the extinction protocol (see below).

Following these remarks, on day two of this study, participants solved simple arithmetic equations, such as “2 + 3/3.” This task is not directly connected to the (supposed) verbal‐skill‐based task of the previous day but is linked to it nonetheless, as again, participants have a limited amount of time to solve these equations. To then allow for the comparison of distinct intervention protocols, time limits for each equation were manipulated differently for each type of experimental/control group. While the reconsolidation group is first presented with unsolvable trials, before then going on to experience demanding, but solvable equations, the extinction‐based control group experiences solvable equations from the start. As a result, one may argue that the extinction‐control group may experience their success in this task as not directly connected to their failure on the previous day, as participants may undergo discrimination learning by experiencing high mathematical abilities in one task and low verbal abilities in the other, instead of thinking of the previous failure in regard to a global disability (which would reflect the classical helplessness attribution style). Thus, this group may experience extinction but not reconsolidation, as this represents new learning rather than interference with the memory trace formed on day one. Conversely, in the reconsolidation group, which at first (again) experiences inability to solve the task at hand, a clear connection to the previous day should be perceived as both tasks highlight their insufficient processing speed. Later, when the task becomes easier (unnoticeably to the participants), this group should then experience an expectation error before the extinction protocol takes effect.^2^

### 2.6 Psychophysiological Measures

Finally, the paradigm presented above needs to be extended to include psychophysiological measures, as previous research indicates that semantic memory about the associative link between UCS and CS is not directly erased. Soeter and Kindt (2015) report that spider phobics, who completed a successful reconsolidation‐based extinction training, stated that they were still afraid of spiders, albeit failing to show a corresponding physiological response. This suggests that the explicit knowledge about their fear was not changed, while their implicit physiological reaction was successfully altered. This suggests that the reconsolidation‐specific effects on day 3 may not be detectable by explicit self‐ratings but rather by behavioral and especially psychophysiological measures.

In the context of depression, peripheral physiological stress‐associated measures such as heart rate or electrodermal activity, as well as neural measures including EEG theta responses in midline electrode positions, are suitable candidates for analysis. Numerous studies have demonstrated that EEG‐based midline theta is a reliable correlate of central LH‐related outcomes. Along these lines, theta (4–8 Hz) at frontal midline electrode positions (Fz, FCz) has classically been associated with deployment of cognitive effort (e.g., Arnau et al. 2024; Cavanagh and Frank 2014; Wascher et al. 2014) but also anxious responses (e.g., Cavanagh and Shackman 2015; Osinsky et al. 2017), and feedback processing (e.g., Fu et al. 2023; Trujillo and Allen 2007; also see Rawls et al. 2020), which has often been reported to be distorted in MD patients (e.g., Keren et al. 2018). Theta at posterior midline positions (Pz), on the other hand, has been shown to indicate control perception and helplessness (Forster et al. 2023; Reznik et al. 2017). Additionally, Reznik and colleagues further investigated the index *PFTA* (posterior versus frontal theta activity) by subtracting absolute theta power at electrode position Pz from theta power at Fz as a correlate in an LH‐task. The authors as well as others (Wacker et al. 2006; Walden et al. 2014) mainly discuss this index as a correlate of (approach) motivation, which was decreased in helpless participants. Against this background, the present study focuses on midline theta measures as its main physiological outcome of interest. To this end, PFTA (theta at Pz—Fz) is considered with respect to its supposed relation to motivation, while theta power at electrode positions Fz and Pz is also investigated individually based on their connection to cognitive effort and control perception, respectively.

### 2.7 Reconsolidation Facilitation

To further improve the possible effects (sizes), following the intervention protocols on T2, the intervention was followed by a brief ergometer training session shortly afterward. On this note, Keyan and Bryant (2017b) were able to show that this method is suitable for strengthening an already consolidated memory, which is in line with various studies that show physical exercise to increase learning effects (also reconsolidation‐based learning; e.g., Keyan and Bryant 2017a, 2019; Loprinzi et al. 2019) and follows the idea that updating in the RW may either in or decrease the strength of an association based on which information is presented. Further, this training standardizes the experience in the post‐reconsolidation‐interference phase, which may decrease variance between participants and prevent further interference by following experiences and information.

## 3 Existing Data

The study presented here was originally planned and started in 2019. Due to the Sars/Cov‐II pandemic that emerged at that time, the study could not be carried out past the point of 16 participants. Nonetheless, this pilot investigation largely followed the same procedure as is shown below. As discussed in the following sections, sample size calculations and formulation of our hypotheses were based on this data. Details, including analyses in EEG and rating data obtained from this sample, can be reviewed in Section 2 in the Supporting Information. The data were not included in the final analysis of this registered report. A notable takeaway from these analyses was that, conversely to results reported by Reznik et al. (2017), we found a negative correlation between motivation and PFTA. However, the reconsolidation group showed more pronounced decreases in PFTA across measurement occasions as compared to control groups, indicating more advantageous results.

## 4 Hypotheses

This study seeks to evaluate a reconsolidation‐based intervention protocol in the context of depression, operationalized via LH. This helplessness should be present in behavioral, motivational, cognitive, and psychophysiological data. Thus, the following three hypotheses each target some of these domains as dependent variables (DVs).

Hypothesis 1a *In wrongly answered (no answer or wrong solution) trials without a time limit, RT decreases from measurement occasion T1 to T3. This indicates faster giving up (i.e., a behavioral LH response). Accordingly, RT should decrease less in the reconsolidation group than in the other groups*.

Hypothesis 1b *Across all trials, we hypothesize that the overall number of solved anagrams across measurement occasion T1 and T3 is preserved most in the reconsolidation group. Accordingly, the number of solved trials should decrease less in the reconsolidation group than in the others*.

Hypothesis 2 *PFTA decreases more between T1 to T3 in the reconsolidation group than in the other groups, representing an electrophysiological correlate of motivation (a cognitive‐affective variable related to LH)*.^3^

Hypothesis 3 *Self‐rated motivation and the feeling of doing better and better within the test day (a variable indicating favorable cognition that opposes LH) will be maintained more strongly from T1 to T3 in the reconsolidation group than in the other groups*.

## 5 Method

### 5.1 Sample

Eligible for participation were individuals who were at least 18 years old and were not currently depressed (measured via the BDI‐II, cut‐off: 20), had no history of depression or other psychiatric disorders (measured via a screening question), and showed no signs of suicidal ideation (indicated by the corresponding BDI‐II item with a cut‐off of 1). Also, all participants need to be German native speakers and physically capable of completing ergometer training. Furthermore, blood‐ or syringe‐phobic individuals were excluded. Finally, individuals with hairstyles that are not suited for EEG application (e.g., dreadlocks) could not participate in the study.

All participants were recruited on a platform provided by the Julius‐Maximilians‐University Würzburg. Participants received monetary compensation or course credit. All participants provided written consent after reading a detailed description of the study and the collected data. This study was reviewed and approved by the local ethics committee, given that participants were subject to a detailed and standardized debriefing at the end of the experiment.

### 5.2 Sample Size Calculation

**FIGURE 1:**
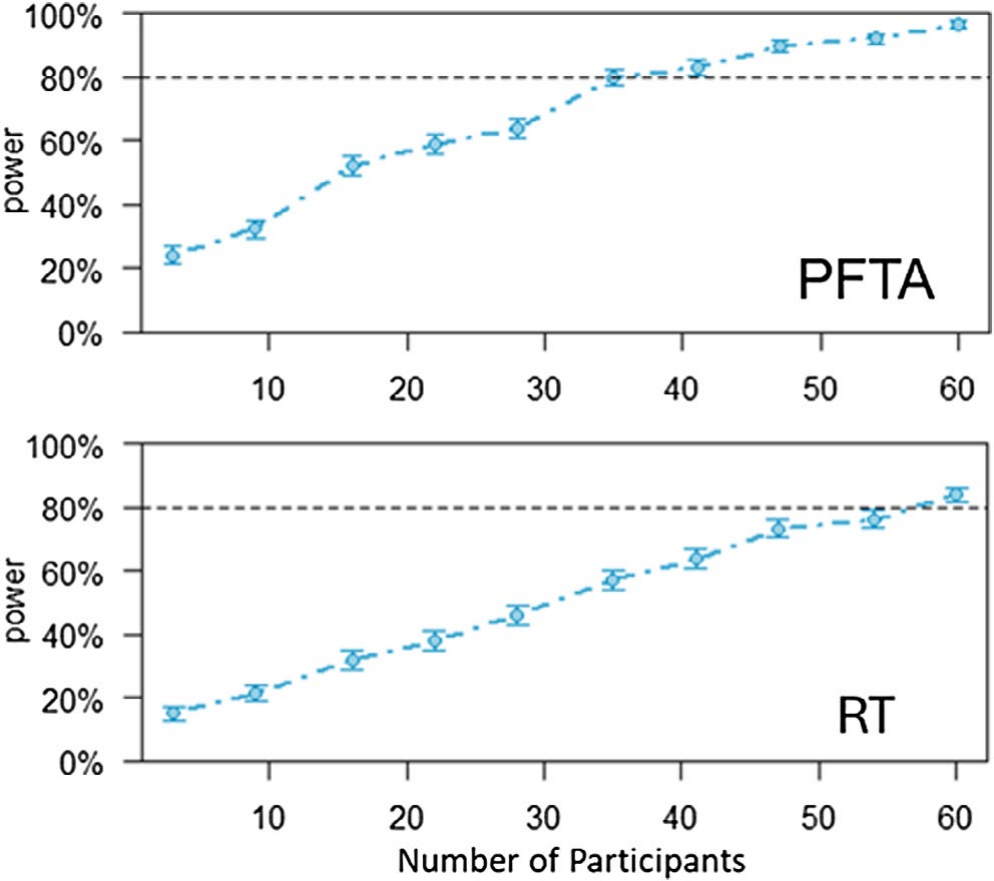
Estimated power to find a significant interaction between measurement occasion and group based on Monte‐Carlo simulations by the “simr” package.

Based on a small sample of pilot data, multilevel mixed models (MLMMs) were fit, and a subsequent sample size calculation was carried out via Monte‐Carlo simulations based on these results. The simulation and power estimation were carried out via the R (R. C. Team 2013) package “simr” (Green and MacLeod 2016). The sample size was calculated for the level 2 within‐between interaction term of measurement occasion (T1 vs. T3) and group (Reconsolidation vs. Extinction, vs. Reactivation) with RT and PFTA in trials without limit restriction as DVs. These effects were chosen as they may be the hardest to find (since only 10 trials at each measurement occasion can be analyzed). Figure 1 illustrates that a power of at least 80% was achieved for both effects if a sample size of 60 participants was used (see Figure 1). As a result, this study sought to recruit participants were until 60 full and eligible data sets were collected.

### 5.3 Outliers and Data Exclusion Criteria

Data that was more than 3.29 standard deviations away from the grand mean were deemed as outliers (Tabachnick and Fidell 2013). Since MLMMs are based on maximum likelihood estimates, participants could still be part of analyses even though their datasets may enclose missings due to outlier exclusion. However, if a participant lacked so much data that no reliable estimate is possible, the participant was excluded from the experiment entirely. This was the case if more than 30% of relevant datapoints comprised missings/outliers in either EEG‐based or RT‐related analyses (on a single‐trial level). Furthermore, single trials were excluded within the EEG preprocessing pipeline due to the criteria given below (see Section 5.15).

Additionally, participants and single data points may be excluded due to hints toward insufficient data quality or extremely invariant responses. This includes:

- Ending each trial within 1 s throughout all trials on each test day (doing so after showing effort to complete the task at first would be acceptable) leads to the exclusion of the participant.
- Solving more than 40% of anagrams leads to the exclusion of the participant since no sufficient helplessness may be induced.

All excluded participants and the respective reasoning behind this decision will be listed after data collection. To further prevent visible task disengagement (e.g., closing one's eyes or looking away from the screen), participants were constantly monitored by the experimenters through a webcam. Also, cheating attempts were stopped immediately without excluding the participant.

### 5.4 General Outline of the Procedure

**FIGURE 2:**
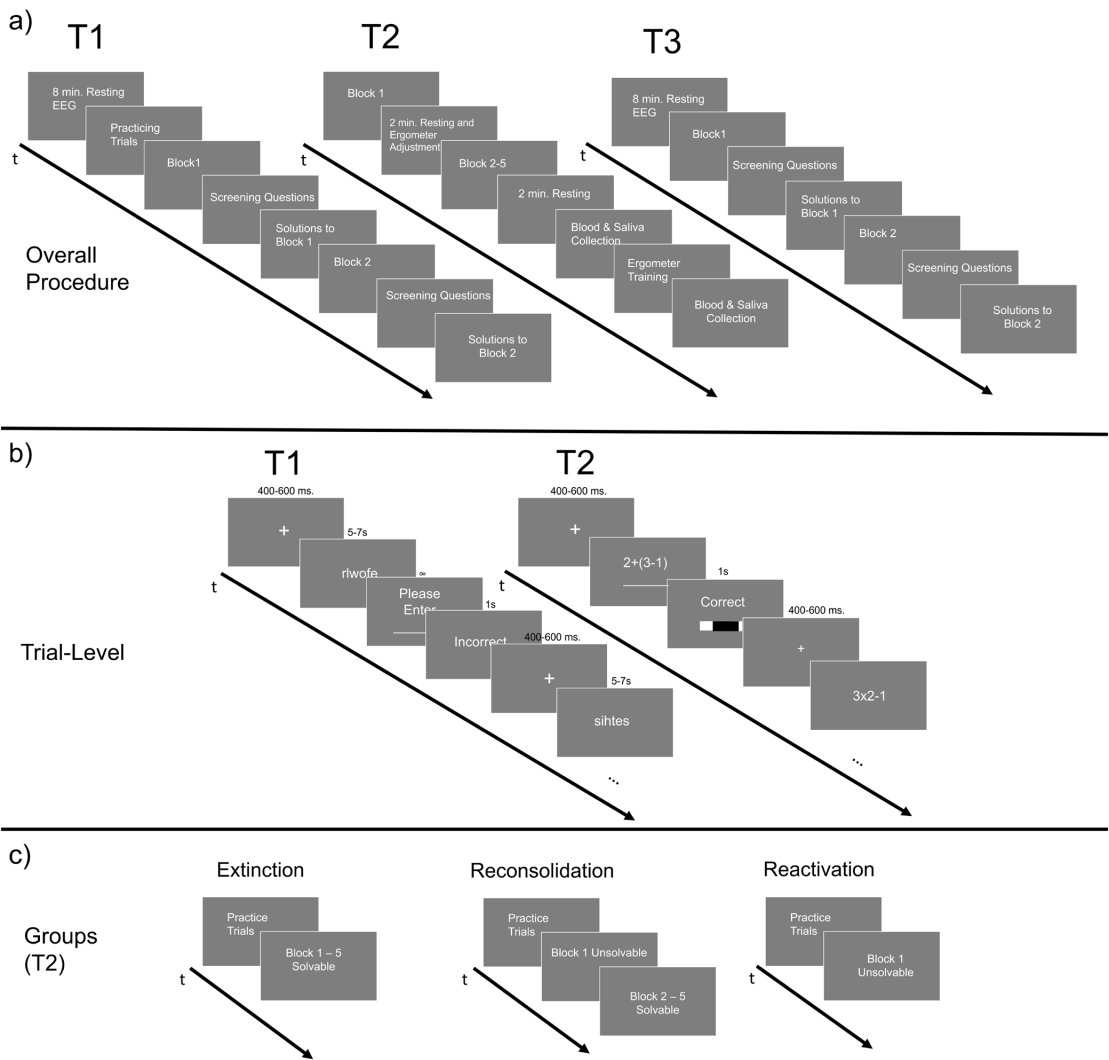
(a) Schematic representation of all three measurement occasions. (b) Schematic representation of trials within the given tasks. (c) Schematic representation of the groups.

The overall experimental setup follows the remarks made above. A schematic illustration can be found in Figure 2a. More details can be reviewed in Section 1 in the Supporting Information. While the first two measurement occasions took place on consecutive days, the third measurement occasion took place two days after the second test day in order to increase the consolidation of the intervention‐related information of T2. Different experimenters supervised each of the three test days so that neither experimenter on T1 nor T3 knew of the participants' group affiliation (assigned at T2). Additionally, the procedure at T2 was conducted in a different laboratory than T1 and T3 were located in, possibly diminishing context retrieval of helplessness beliefs. In sum, this study thus follows a double‐blind, three‐day, three‐group randomized controlled study design.

### 5.5 Online Survey (T0)

Before the experiment began, participants completed an online survey containing items of the BDI‐II and the Big Five Aspect Scale (BFAS; Mussel and Paelecke 2018). Further, in addition to some sociodemographic questions, participants then moved on to complete a short version of the arithmetic task at T2, assessing each participant's individual solving speed (participants were not aware that this task would be relevant at T2). In this online version of the task, participants saw one equation at a time. Once the solution (a number between 0 and 9) was entered, the next equation was presented automatically. To ensure reliability of the data, participants first completed three test trials. Following this, 10 trials were completed. Based on the RT in correctly solved trials, the median solving time was calculated. All data that were not more than 1.5 s above or below the median were then used to calculate the average solving time, which was used as the starting value on T2 (see below). After completing this online survey, participants completed the following experimental protocol.

### 5.6 Procedure at T1 and T3

The procedure at T1 and T3 is identical and follows the schedule below (see Figure 2b). Throughout the entire task on these measurement occasions, participants were recorded via EEG, ECG, and electrodermal activity measures. Furthermore, participants were monitored via a webcam above the task‐presenting monitor.

#### 5.6.1 Resting EEG

At each of these measurement occasions, first, 8 min of resting EEG were collected, including 4 min with closed eyes and 4 min with open eyes. The instruction to open or close the eyes was given every 60 s via audio commands (following either of these orders: OCCOOCOC or COOCCOCO; with O = eyes opened and C = eyes closed).

#### 5.6.2 Anagram Task

Following the resting EEG, the anagram task began. Only on day 1, three solvable practice trials were completed. Then, five blocks of 10 anagram trials each were presented. While all anagrams were per se solvable, most of them were not presented long enough for the participants to solve them (anagram length: 7–10 letters, time limit: jittered between 3 and 6 s).

Each trial started with a fixation cross (jittered between 400 and 600 ms), after which the anagram appeared. Then, after the time was up or the participant ended the trial actively, the participants were asked for the solution to the preceding anagram. Here, no time limit was imposed.^4^ After confirming the entered solution, again a fixation cross appeared (jittered between 400 and 600 ms) before providing written feedback on their answer (“Correct” or “Incorrect,” presented for 1 s, see Figure 2b).

In total, 50 anagrams were presented each day (+three test trials on T1). Each test day, 40 anagrams were presented with a jittered time limit (5–7 s) while 10 anagrams were presented without time constraints, thereby allowing for the assessment of how much effort participants were still showing throughout the task (either how much time they invested before giving up or how long it took to solve the anagram).

By jittering the time constraint in the other trials, participants could not immediately recognize a trial without time limits. Existence of these unlimited anagrams was explicitly announced during the instructions but not signaled before presentation in the upcoming trial. At the end of each block, eight screening questions (see Table 1) were presented. Finally, to show subjects that all anagrams were solvable, thereby preventing external attribution, solutions of the anagrams were presented at the end of each block.

**TABLE 1:** Four dimensions are measured via eight questions.

Item	Scale	Direction
I am disappointed with my performance	Satisfaction with performance	Inverted
I am worse at solving the anagrams than I was at the beginning of this day	Increase in performance over time	Inverted
My motivation to complete the task is high	Motivation	
I am better at solving the anagrams than I was at the beginning of this day	Increase in performance over time	
I believe to be better than average in this task	External attribution	
I am satisfied with my performance	Satisfaction with performance	
I believe to be worse than average in this task	External attribution	Inverted
My motivation to complete the task is very low	Motivation	Inverted

*Note:* To compute the final measure used in analyses, the score from the inverted item is subtracted from the score of the remaining question. If a participant thought of their failure as being better than the average, this implies the belief that the task is too hard for most people. Thus, these questions allude to the idea of external (task‐attributed instead of person‐attributed) attribution of performance deficits. Since external attribution and satisfaction with one's performance may indicate coping mechanisms, opposing LH, these two dimensions may act as covariates rather than dependent variables of helplessness.

Different sets of anagrams were presented at T1 and T3. However, on average, the length of anagrams within each block was held constant across test days. Further, all anagrams without time constraints had the exact same length. These unlimited anagrams were fixed to a certain position within the trial succession across participants.

### 5.7 Procedure at T2

On day 2, participants visited a different laboratory than on T1 and T3; this time solving equations (two arithmetic operations per trial, e.g., 2 + 3/3) instead of anagrams. As stated above, this task was manipulated to produce three groups based on a randomized selection: a reconsolidation group, an extinction group, and a reactivation group. While the time limit for a given trial was fixed at T1 and T3, at T2, the limit was calculated dynamically on a trial‐by‐trial basis, building on the following steps: During the online prescreening, equations without a time limit were administered, allowing for an individually estimated average solving speed for each participant. Based on this measure, in block 1 of T2, then, depending on the group, either 25% was subtracted or added. As a result, each participant started with an individual time limit that was designed to induce the feeling of either “just not being fast enough” or “just being able to make it” (e.g., 2.4 s + 0.6 s = 3 s). This approach was adapted and reprogrammed in PsychoPy after the Montreal Imaging Stress Task (MIST; Dedovic et al. 2005). After entering the solution, subjects again received feedback with the words “Correct” or “Incorrect” for 1 s. Furthermore, in parallel, they received graphical performance feedback through a bar at the bottom of the screen indicating their current performance (see Figure 2b). By introducing this, slightly better‐than‐performance feedback was given in the extinction phase to facilitate positive experiences with the task, even though failure may sometimes occur.

Just like at T1 and T3, participants were presented with 50 trials nested within five blocks. After block 1, participants were asked to rest and relax for 2 min. Afterward, the experimenter guided them to an ergometer located in the same room, asking participants to adjust it to their individual height and needs. This procedure was used to stall the participant for some time, so the RW could open, as it has been shown to open approximately after 10 min. following reactivation (e.g., Monfils et al. 2009; Johnson and Casey 2015).

#### 5.7.1 Ergometer Training

Participants then moved on with the remaining blocks. After completion of all trials, participants then engaged in a short ergometer training (intensive cycling at 70 W + 40 W/5 min) that was used to amplify the preceding learning within the arithmetic task and to provide a period without other interfering information (thus, increasing effect sizes). Regardless of the group, this procedure was the same for all participants. To further investigate the influence of cortisol and blood‐BDNF (brain‐derived neurotrophic factor) as potential covariates of interest, saliva and blood samples were collected before and after the training. BDNF has often been shown to convey learning in various experimental tasks (e.g., Bekinschtein et al. 2014), stimulating consolidation and reconsolidation generally (e.g., Cunha et al. 2010; Radiske et al. 2015) and following an ergometer training specifically (Keyan and Bryant 2017b, 2017a).

### 5.8 Experimental and Control Groups (T2)

The task at T2 is designed to provide groups with distinct experiences. As stated above, the reconsolidation group ought to receive a reminder of their experience at T1 before an extinction protocol is administered (see Figure 2c). Thus, for this particular group, the arithmetic task worked as follows: First, the average time needed to solve the practice equations was calculated. Then, 25% of this time was subtracted before the first equation of block 1 was presented. Further, within block 1 if a participant solved two equations in a row, the time limit was again decreased by 10%, ensuring that participants failed considerably often. Then, the above‐mentioned 10‐min break took place during which participants were asked to rest and adjust the ergometer. Subsequently, blocks 2–5 were completed. During these blocks, to give participants a positive experience, 25% of the time was added to the estimated average solving speed as base value. If a participant failed to solve two equations in a row, the limit was again increased by 10%. This procedure proceeded until the task was completed. In sum, this group should first experience insufficiency before moving on to experience an “I made it just in time” effect.

The extinction group, on the other hand, only follows the procedure that ought to provide a positive experience. This group thus differs from the reconsolidation group due to a lack of a reminder in block 1; i.e., participants within this group started with 25% increased average time limits from the start.

Finally, the reactivation group only experienced the reminder block (block 1 of the reconsolidation group) before moving on to the ergometer training (still, the 10 min. waiting time after block 1 was also completed in this group). As a result, this group was only presented with one block of the task, instead of five, as the other groups were.

### 5.9 EEG Collection

EEG data was collected via a passive 64‐electrode setup by EASYCAP that follows the 10–10 system and uses Afz as ground, while employing Cz as online reference. The setup can be reviewed in Section 5 in the Supporting Information. The sampling rate was set to 500 Hz. During recording, a high‐pass filter of 0,1 filter was imposed. The signal was processed via amplifiers by BrainVision and recorded by the BrainVision Recorder.

### 5.10 Self‐Rate Measures

At the end of each block at T1 and T3, the solutions of the previous anagrams are presented to the participants to assess their subjective experience. Before this, a total of eight questions were presented on visual analog scales with poles “not at all” and “very much.” The questions are summarized in Table 1. In total, screening questions were intended to indicate four different constructs, with two questions indicating one construct respectively (also see Table 1). The order of questions was randomized after each block. Furthermore, as stated above, the BDI‐II and the BFAS were administered in an online survey preceding the experiment.

### 5.11 Material

All experimental tasks were programmed in Psychopy version 2022.2.4 (the pilot studies were programmed in version 3.2.4; Peirce et al. 2019). The anagrams and equations used in the tasks were custom‐made.

Psychophysiological measures include EEG, ECG, and electrodermal responses, even though the current study focuses on EEG‐based measures. All analyses were carried out via the lme4 R package (Bates et al. 2014) and MATLAB.

### 5.12 Analyses: EEG Preprocessing

EEG preprocessing was carried out following the MATLAB‐based EPOS pipeline (Rodrigues et al. 2021). This pipeline mainly involves the following steps: First, the data was re‐referenced to an average reference. Subsequently, channels of low quality were excluded and interpolated based on surrounding channel data. The data was then epoched into pieces of 2.5 s length (−300 to 2200 ms). Following this, the data was high‐pass filtered (1 Hz) before an ICA was performed. Then, segments of low quality were excluded before a second ICA was performed (only if segments were deleted from the data). To ensure reproducibility, ICA components were then automatically rejected via ADJUST and MARA using SASICA. In the next step, the data was CSD‐transformed via the package provided by (Kayser and Tenke 2006) before Morlet wavelets (fixed cycles of 3.5 s, log spaced) were used to extract the power of frequency bands (4–8 Hz for theta band power). Finally, the power measures were then normalized via natural logarithms. To compute PFTA, absolute theta power at Fz was subtracted from Pz. The time window for theta power extraction was set to the first 2 s after stimulus onset.

### 5.13 Analyses: General Multilevel Modeling Approach

The overall approach to the MLMM of trial‐by‐trial data utilized in this study follows a data‐driven approach that determines the best‐fitting model to the given correlation‐covariation matrix before investigating a priori described fixed effects. To do so, for all the following statistical analyses, first, an empty random intercept model was fit. Then, a random slope is added for the “trial”‐variable. In the next model, fixed‐effect estimates for either “group” or “number of successful trials in block 1 at T2,” as well as the measurement occasion and their respective interaction term were introduced into the model. Finally, in the last model, covariates that may introduce substantial error and bias into the model if not included were introduced (only their main effects). The best‐fitting model was determined using the corrected Akaike Information Criterion (AIC) and the probability of information loss (Burnham and Anderson 2002). As a result, concerning each hypothesis, the following models were fit, yet only the best‐fitting model was interpreted:

- 1. An empty random intercept model.
- 2. An empty random intercept, random slope model.

Depending on the significant contribution to model fit by the random slope, either model 1 or model 2 was used for the inclusion of further variables in models 3 and 4:

- 3. *Measurement occasion*, *group*, and their interaction were introduced as fixed effects.
- 4. *Measurement occasion* and *number of successful trials in block 1 of T2* were introduced as fixed effects.
- 5. The *trial*, *external attribution*, and *satisfaction with performance* are introduced as covariates. Even though no meaningful three‐way interaction between *measurement occasion*, *trial*, and *group/successful trials* was found in the pilot data, this three‐way interaction term was still introduced into the model due to theoretical accounts (see above). *External attribution* and *satisfaction with performance* are included only as main effects.

Since the following hypotheses target the comparison of the reconsolidation group with the control groups, a *simple contrast* was chosen for group comparisons with the reconsolidation group as the reference category. Other contrasts may not be valid, since the control groups may not show similar effects, which opposes contrasts that aggregate both groups to form the reference level.

#### 5.13.1 Analyses: Hypothesis 1a

In trials that have no time restriction, RT was extracted and used as the dependent variable in the models described above. However, only trials that ended in a false anagram solution, including those without any attempt to solve the anagram, were taken into account. By doing so, RT can be interpreted as an indication of how much time participants were eager to invest before giving up. We expect a significant interaction of measurement occasion and group (successful trials in block 1 of T2) pointing toward less pronounced reductions of RT across measurement occasions for participants who were in the reconsolidation group (or had less successful trials at block 1 of T2). In this analysis, the reactivation group was excluded.

#### 5.13.2 Analyses: Hypothesis 1b

In line with this, we expected higher rates of solved anagrams in the reconsolidation group as opposed to the others on T3. Thus, once more, a significant interaction of measurement occasion and group (number of successful trials in block 1 of T2) was anticipated. Regarding this calculation, a logistic generalized MMLM approach used the correctness of answers as the dependent variable, while the independent variables followed the approach described above.

#### 5.13.3 Analyses: Hypothesis 2

Theta activity at Pz minus Theta activity at Fz was calculated to form PFTA. This was then used as the dependent variable in the models described above. We hypothesized a significant interaction of measurement occasion and group, with greater decreases over measurements in the reconsolidation group.^5^

#### 5.13.4 Analyses: Hypothesis 3

Self‐rated motivation and the feeling of doing better in solving the anagrams are used as DV in the additional two models that follow the same procedure as described above. Again, a significant interaction of measurement occasion and group (successful trials in block 1 of T2) was anticipated and thought to show preserved motivation and feeling of doing a decent job in the task for the reconsolidation group.

#### 5.13.5 Analyses: Alpha‐Error Accumulation

In total, regarding each hypothesis, 3 post hoc tests were computed comparing the contrasts T3–T1: reconsolidation‐extinction, T3–T1: reconsolidation‐reactivation, and T3–T1: successful trials. These statistics are reported in each hypothesis. As a result, the corresponding *p*‐values are reported and interpreted against a within‐hypothesis Bonferroni‐Holm correction against error cumulation of the three respective tests.

## 6 Results

### 6.1 Deviations From the Preregistration

Due to unforeseen levels of line noise in the EEG data, the preprocessing was adjusted to include appropriate Notch filters (50, 100, 150, 200, 250 Hz; details can be reviewed in the supplemental MATLAB code). Further, due to a technical issue, only 49/50 anagrams at T1 were recorded, and in one participant, some anagrams were easier than for the others at T1.

### 6.2 Sample Characteristics

The final sample consists of 60 participants (63.3% women) with a mean age of 26.15 (SD: 9.24). On average, the sample showed low BDI‐II scores (mean = 4.35, SD = 4.05; further details can be reviewed in the Section 3.1 in the Supporting Information).

### 6.3 Manipulation Check

First, results concerning the self‐rate data and behavior on T1 are presented to ensure that the experimental manipulation successfully induced helplessness. Details on the analyses can be reviewed in the R‐Code and Additional Information provided in Sections 3.2 and 3.3 of the Supporting Information.

#### 6.3.1 Probability of Failing (Behavioral Data)

At T1, participants on average failed to solve 92.46% of anagrams within a time limit and 60.37% of times in anagrams without a time limit. Taken together, on average, 6.58 out of 49 anagrams (including those without a time limit) were solved at T1.

**FIGURE 3:**
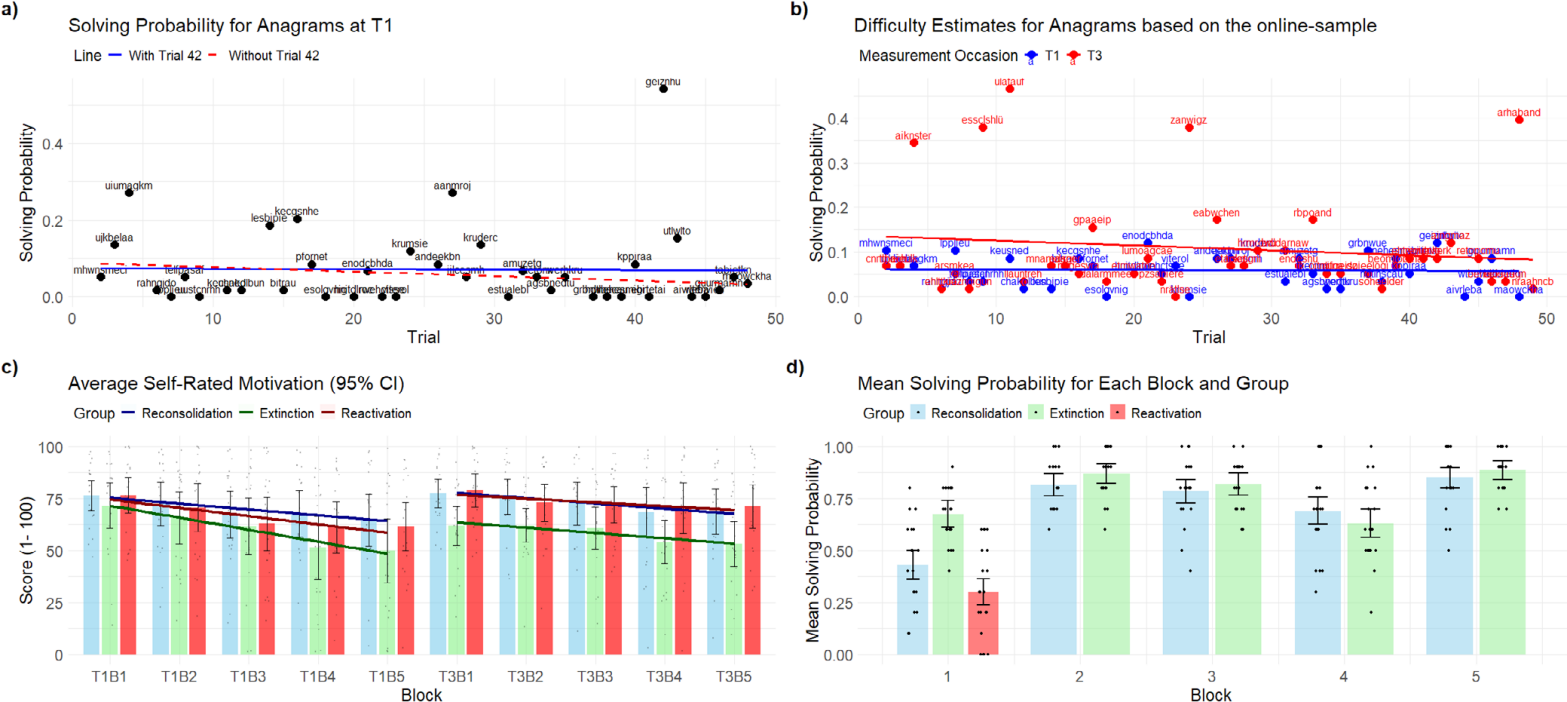
Illustration of manipulation check results. (a) This plot depicts the overall solving probability of time‐restricted anagrams at T1 for the complete sample. The blue solid line indicates a linear regression line, that is distorted by trial 42, which seems particularly easy. Excluding this trial leads to a steeper slope (red line), indicating a highly significant decrease in solving probability over time. (b) Estimated difficulty scores based on the percentage of individuals who solved the anagram within the time window of the main study. Note that the online sample did not experience any time restriction but were simply asked to solve the anagram as quickly as possible. Though T3 held five anagrams that were particularly easy, no systematic slope in difficulty within test days was present. (c) Average self‐rate measures for motivation, showing significant decreases in motivation across both T1 and T3. (d) Mean solving probability for equations at T2, indicating higher success rates for the extinction group (as intended) as compared to the others. In blocks 2–5, success rates were similar across extinction and reconsolidation groups.

Contrary to our expectations, the probability of failing did not linearly increase over time (*p* > 0.2). Figure 3a illustrates that this finding may be the result of a confound between trial number and item difficulty, indicating the anagram in trial 42 to be especially easy. After removing this trial from the analysis, a linear decrease in solving probability emerged (*z* = −3.304, *p* < 0.001). However, to further rule out that this effect was itself the result of unintended increases in item difficulty in later trials, item difficulty estimates were collected from an additional online study featuring 58 participants, who were presented with the anagrams in random order and without any time restrictions (see Figure 3b). Notably, the decrease in solving probability over time remained robust after including difficulty estimates from the online sample as a covariate (*z* = −2.774, *p* = 0.006; for more details, see Section 2.2 of the Supporting Information). Conversely to these results in time‐limited trials, participants did not show any linear trend toward increases or decreases in time before giving up in nonrestricted erroneous trials (*p* > 0.1).

Overall, these results indicate that participants experienced substantial failure to solve the task while showing hints of decreasing effectiveness in solving anagrams over time, which is generally in line with LH.

#### 6.3.2 Self‐Rate Data

In line with the theoretical background of LH, self‐rated motivation decreased over time (*t* (59) = −5.709, *p* < 0.001), resulting in an average rating decrease from 74.8 in block one to 58.6 in block five (scale 1–100). This decrease did not significantly differ between groups (see Section 2.3 in the Supporting Information), which is expected since randomization did not take place before T2. Importantly, the participants consistently reported attributing their performance issues to internal factors as participants of all groups estimated their performance to be worse than that of others (grand mean: 21.95 on a scale from 1 to 100). This attribution did not change throughout the blocks at T1 (*p* > 0.3). In the same vein, participants indicated low satisfaction with their performance (grand mean: 28.84 on a scale from 1 to 100), which again, did not change over time (*p* > 0.2). Finally, participants indicated that they were not becoming more successful in solving the anagrams over time (grand mean: 33.58 on a scale from 1 to 100). This rating decreased significantly over time (*t* (59) = −2.741, *p* = 0.008) though the effect seems to be driven mainly by relatively high values in block 1, which may be owed to the way the question was formulated (asking whether they felt they were doing better over time, which may be hard to estimate after only 10 trials in the first block, and may stimulate ratings near the scale's center for the first block).

Taken together, participants rated their motivation to decrease significantly over time, while satisfaction with their performance was low, and attribution of their failure was stated to be caused by internal rather than external factors. Furthermore, participants seemed to show decreased efficacy in solving anagrams over time. Overall, these results indicate successful manipulation of helplessness‐related parameters at T1.

#### 6.3.3 Randomization at T2

At measurement occasion T2, the randomization process should have led to significantly more failed trials in block one for the reconsolidation and reactivation groups than in the extinction group. Indeed, this effect could be found (reconsolidation—extinction: *t* (57) = −4.137, *p* < 0.001; reactivation—extinction: *t* (57) = −6.332, *p* < 0.001). Subsequently, the rate of failure was meant to be equal across the reconsolidation and extinction groups in blocks 2–5, which was also evident (reconsolidation—extinction in blocks 2–5: *p* > 0.7).

In line with this, rating measures concerning the feeling of improving over time differed across groups in block one (reconsolidation—extinction: *t* (57) = −2.501, *p* = 0.015; reactivation—extinction: *t* (57) = −2.092, *p* = 0.041), but not throughout blocks 2–5 (reconsolidation—extinction in blocks 2–5: *p* > 0.7). Similarly, rating differences on dissatisfaction with one's performance were more significant in block one (reconsolidation—extinction: *t* (57) = −2.586, *p* = 0.012; reactivation—extinction: *t* (57) = −2.371, *p* = 0.021) than in blocks 2–5 (reconsolidation—extinction in blocks 2–5: *t* (57) = −2.083, *p* = 0.044). Importantly, the same is true for the participant's feeling of doing better in the task than others would (block 1: reconsolidation—extinction: *t* (57) = −2.903, *p* = 0.005; reactivation—extinction: *t* (57) = −2.045, *p* = 0.045; blocks 2–5: reactivation—extinction: *t* (57) = −1.939, *p* = 0.06), implying that participants attributed task outcomes as a correlate of their performance rather than changes in task difficulty. Otherwise, no group differences in motivation ratings were found in block one (reconsolidation—extinction: *p* > 0.7; reactivation—extinction: *p* > 0.4) or blocks 2–5 (reconsolidation—extinction: *p* > 0.7). In sum, these results indicate that the randomization process successfully manipulated the experience of failure, thereby supposedly influencing the reactivation of helplessness.

### 6.4 Registered Hypotheses Results

#### 6.4.1 Hypothesis 1a

**FIGURE 4:**
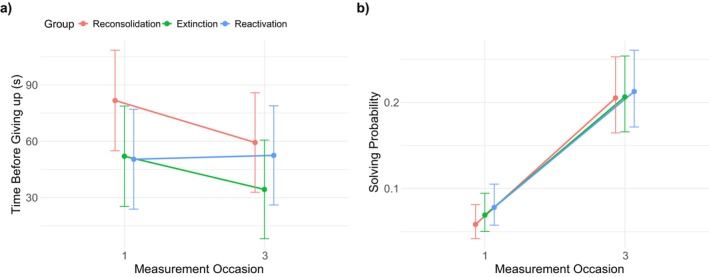
Estimated effect plots for Hypothesis 1a and 1b. (a) Significant differences in groups across measurement occasions regarding the time before giving up on solving a non‐time‐restricted anagram indicate group differences at T1, rather than T3, which contradicts the assumptions. (b) Solving probability did not significantly differ between groups across measurement occasions. Error indicators depict 95 CI.

Following the model selection procedure described above, the optimal model consisted of only a random intercept for each participant and fixed effects for group and measurement occasion (main effects and their interaction). The results indicate that the reconsolidation and extinction groups showed a similar effect regarding the time spent on unrestricted anagrams across measurement occasions (measurement occasion × extinction‐measurement occasion × reconsolidation: *t* (669) = 0.539, *p* = 0.590, *holm* > 0.999), while the reactivation group differed significantly from the reconsolidation group (measurement occasion × reactivation‐measurement occasion × reconsolidation: *t* (669.17) = 2.794, *p* = 0.005, *holm* = 0.015). Figure 4a illustrates that the reconsolidation and extinction group show a trend toward decreasing time spent, while the reactivation group shows no change across measurements.

Substituting the group with the reactivation at T2 (and excluding the reactivation group from the analysis) revealed no significant (linear) influence of the amount of reactivation on the difference in time spent across measurement occasions (*t* (442.805) = −1.124, *p* = 0.261, *holm* ≥ 0.999).

#### 6.4.2 Hypothesis 1b

Regarding the probability of solving time‐restricted anagrams, a similar pattern to the one described in Hypothesis 1a emerged (see Figure 4b). No significant difference between groups across measurement occasions was present (measurement occasion × extinction‐measurement occasion × reconsolidation: *z* = −0.745, *p* = 0.456, *holm* = 0.912; measurement occasion × reactivation‐measurement occasion × reconsolidation: *z* = −1.162, *p* = 0.245, *holm* = 0.735). At T3, the average probability of solving an anagram was generally greater than at T1 (mean effect for measurement occasion: *z* = 8.454, *p* < 0.001), indicating differences in item difficulty across test days (see Section 3.2 in the Supporting Information for an analysis showing that this is true, based on the data from the additional online sample). Again, substituting the group with reactivation in block 1 of T2 showed no significant effect (*z* = −0.651, *p* = 0.515, *holm* = 0.912).^6^

#### 6.4.3 Hypothesis 2

##### 6.4.3.1 Theta at Fz

**FIGURE 5:**
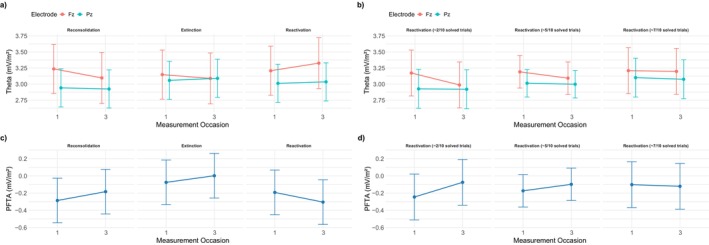
Estimated effects for Hypothesis 2. (a) Estimated effects for electrode positions Fz and Pz regarding the three‐way interaction between measurement occasion and group. Allowing for a random slope of measurement occasion. Without considering the random slope for measurement occasion, theta at Fz significantly decreases more from T1 to T3 as compared to the other groups. After considering this random slope, the effect vanished, though the plot does not considerably change, as effect sizes are not affected by this. (b) A similar pattern emerged when considering reaction at T2 instead of groups. Note that more solved trials indicate less reactivation. (c) Results concerning PFTA, depicting results from the same three‐way interaction, showing increases in PFTA from T1 to T3 in the reconsolidation and extinction, but not the reactivation group. (d) A similar effect to what is depicted in (c) also emerged when substituting group for the number of solved trials in block 1 of T2. Error indicators depict 95 CI.

The model selection procedure described above indicates the superiority of a model featuring only the random intercept for each participant. In line with the hypothesis and the pilot data, there was a significant interaction effect between measurement occasion and group (measurement occasion × extinction‐measurement occasion × reconsolidation: *t* (5800) = 2.717, *p* = 0.007, *holm* = 0.007; measurement occasion × reactivation‐measurement occasion × reconsolidation: *t* (5800) = 8.546, *p* < 0.001, *holm* < 0.001). Figure 5 shows that theta decreases across measurement occasions in the reconsolidation group, remaining rather constant in the extinction group, and increasing in the reactivation group. The best‐fitting model did not include a three‐way interaction with trial, nor main effects for satisfaction or attribution. Substituting *group* with the reactivation at T2 (number of solved trials in Block 1 of T2) revealed that those who experienced more failure in block one of T2 showed larger decreases in theta (measurement occasion × reactivation = *t* (3874) = 5.828, *p* < 0.001, *holm* < 0.001).

##### 6.4.3.2 Theta at Pz

Regarding theta at electrode position Pz, again, the random intercept model (with no random slope for trial) was analyzed. Conversely to the results concerning Fz, no group difference in trajectories across measurement occasions was found (measurement occasion × extinction‐measurement occasion × reconsolidation: *t* (5792.67) = 1.862, *p* = 0.063, *holm* = 0.189; measurement occasion × reactivation‐measurement occasion × reconsolidation: *t* (5794.20) = 1.561, *p* = 0.119, *holm* = 0.238). Additionally, no three‐way interaction between measurement occasion, group, and trial was present, while a highly significant relation to self‐rated attribution (*t* (5846.23) = 4.347, *p* < 0.001) and satisfaction with one's performance emerged (*t* (5833.89) = −3.912, *p* < 0.001). Substituting group with reactivation at T2 revealed no significant effect (measurement occasion × reactivation = *t* (3868.41) = −0.708, *p* = 0.479, *holm* = 0.479).

##### 6.4.3.3 PFTA

Concerning PFTA, again, the random intercept model showed the best fit. Only the reactivation group showed a significantly different progress across measurement occasions when compared to the reconsolidation group (measurement occasion × reactivation‐measurement occasion × reconsolidation: *t* (5797.03) = −6.095, *p* ≤ 0.001, *holm* < 0.001). No effect was present for the extinction group (measurement occasion × extinction‐measurement occasion × reconsolidation: *t* (5793.65) = −0.737, *p* = 0.461, *holm* = 0.461). Further, no three‐way interaction with trial was significant. Substituting group with reactivation at T2 revealed a significant effect (measurement occasion × reactivation = *t* (3868.95) = −5.457, *p* < 0.001, *holm* < 0.001), indicating larger increases over measurement occasions for those who experienced more reactivation.

#### 6.4.4 Hypothesis 3

Analyses concerning the progression of motivation across test days showed a main effect of decreasing motivation (across both T1 and T3: *t* (122.326) = −2.554, *p* = 0.011). However, no significant effect concerning group differences was present (measurement occasion × extinction‐measurement occasion × reconsolidation: *t* (472.61) = −1.727, *p* = 0.085, *holm* = 0.255; measurement occasion × reactivation‐measurement occasion × reconsolidation: *t* (475.11) = 1.175, *p* = 0.241, *holm* = 0.482). Concerning the feeling of improving throughout the task, neither the main effect of block (*t* (163.44) = −1.874, *p* = 0.063), nor the interaction between measurement occasion and group were significant (measurement occasion × extinction‐measurement occasion × reconsolidation: *t* (468.91) = 0.434, *p* = 0.664, *holm* > 0.999; measurement occasion × reactivation‐measurement occasion × reconsolidation: *t* (473.37) = −0.573, *p* = 0.567, *holm* > 0.999). Substituting group with reactivation at T2 also resulted in nonsignificant interactions between measurement occasion and reactivation, for both motivation ratings (*t* (314.23) = −0.774, *p* = 0.440, *holm* = 0.482.) and improvement ratings (*t* (344.89) = −0.955, *p* = 0.340, *holm* > 0.999).

### 6.5 Summary of Additional (Exploratory) Analyses and Results

**FIGURE 6:**
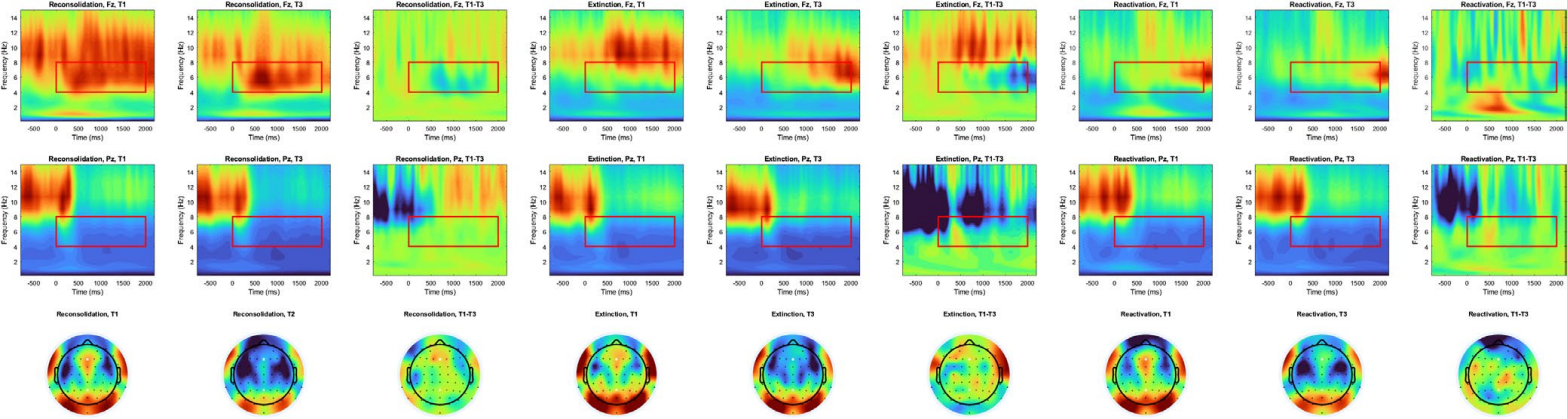
Time frequency plots and topoplots for each group across measurement occasions. Red squares indicate the time and frequency window of interest. Scales were set to mean power values for each electrode across both measurement occasions and all groups ±1 SD.

To ensure that the preregistered analysis approach produced valid and robust results, a number of additional analyses were carried out to critically test for the possibility of unanticipated confounds, misspecifications of preregistered modeling approaches, and unintended production of effects through preprocessing decisions. Details on these analyses can be found in Sections 4.1–4.5 in the Supporting Information. The following overview summarizes the main findings for each analysis:

- Since the largest differences between groups were found for theta at Fz, we investigated whether this measure was related to motivation and performance, as the preregistered hypotheses did not specifically test whether an increase or decrease was related to favorable outcomes. We found that theta at Fz was only then positively related to task performance when alpha power at Fz was also considered in the same model. Theta at Fz was further positively correlated to self‐rated motivation, indicating positive effects in those who showed increases in theta across measurement occasions (see Section 4.1 in the Supporting Information).
- To rule out that group differences were the result of participant variables/properties rather than the manipulation at T2, we tested whether the number of solved equations in block 1 of T2 (which is supposed to be the result of experimental manipulation) was related to trait‐variables of participants and found no significant effect. In combination with the results from the manipulation check (see above), this suggests that the reactivation at T2 was successfully manipulated by the experiment independently of the personality traits of our sample (see Section 4.2 in the Supporting Information).
- To check whether the results concerning theta measures were the result of specific preprocessing decisions, we performed a large multiverse analysis comparing results from various preprocessing pipelines. We did not find any indication of the inappropriateness of the preregistered approach when compared to others. Time frequency plots and topoplots for each preprocessing pipeline are provided at OSF (also see Section 4.3 in the Supporting Information). Figure 6 shows the corresponding overview regarding the preregistered approach.
- Most importantly, reconsidering the preregistered multilevel modeling approach, we noticed that we did not consider allowing a random slope for measurement occasion. Adding this to the model significantly improved model fit across all related analyses (see the supplemental R‐Code for details). While this did not affect the size of effects, CI intervals increased drastically through the related reduction of degrees of freedom, rendering all previously significant effects of Hypothesis 2 insignificant. Importantly, despite diverging from the preregistered analysis by including this term, model comparisons indicate the superiority of this model (for more details, see the discussion below and Section 4.4 of the Supporting Information). Please note that the discussion regarding the appropriateness of including this random slope is rather technical and complex. Though there are arguments for and against its inclusion, the authors advocate for its consideration in the model.
- Finally, as briefly mentioned above, blood samples were collected from participants after completing the intervention protocol at T2. Including BDNF levels in the models mostly leads to the reemergence of the originally significant results regarding midline theta. However, since this analysis was not preregistered and due to BDNF levels being modeled as a between‐variable, these results can only indicate BDNF as an interesting covariate for future investigations but not confirmatory evidence (see Section 4.5 in the Supporting Information for details).

## 7 Discussion

This registered report utilized a LH task to investigate the benefit of reconsolidation‐based interventions in the context of MD. To do so, an LH‐task was administered at two different measurement occasions (T1, T3), while three different interventions were applied in a randomized controlled, double‐blind setting in between sessions (T2). Along this line, the present study assumed that helplessness beliefs result from a conditioning process that can be reactivated based on the contingency between cognitive effort and outcome probability.

### 7.1 Manipulation Check

Generally, the LH‐task (T1, T3) worked mostly as intended. Results of the manipulation check show that participants largely failed throughout the task, performing worse over time. In line with this, participants reported a considerable decrease in motivation throughout the task while robustly indicating that they felt like they were doing worse than others would (implying internal attribution of their failure). Furthermore, ratings robustly indicated low satisfaction with their performance and the feeling of being unable to improve over time. All in all, these results speak to LH‐congruent decreases in motivation and cognitive effort while also implying LH‐congruent beliefs and negative self‐appraisal. Furthermore, both performance and rating data indicate that the randomization process at T2 worked as planned; participants in the reactivation and reconsolidation group showed significantly more failures in block 1 of T2. The reconsolidation group then went on to show a similar performance to the extinction group. Self‐appraisal ratings similarly differed at first across groups in block 1 of T2 before becoming similar in subsequent trials. Throughout T2, satisfaction and attribution ratings correlated to task performance, implying that participants attributed both positive and negative outcomes internally.

### 7.2 No Superiority of the Reconsolidation‐Based Protocol

Conversely to our hypotheses, neither self‐rating, EEG measures, nor behavioral variables changed with respect to differences in interventions applied at T2. Interestingly, both motivation and performance were not only similar in the reconsolidation and extinction group (implying a lack of superiority of either intervention protocol) but also across the reconsolidation and reactivation group. Since the reactivation group did not receive any explicitly planned positive experience at T2 but instead went on to suffer failure a second time, this result elicits further questions. As can be seen from the large impact of random effects across many of our analyses (for details on specific analyses, please review the additional information in the Supporting Information or the R‐Script), we found considerable interindividual differences in motivation ratings, performance indices, and their respective progression throughout the experiment, which were not related to the experience at T2. Along this line, one may argue that both motivation and performance are subject to large interindividual traits that outweigh the influence of our intervention protocols. This interpretation would be in line with studies showing trait‐like differences across patients, determining both the short‐term efficacy and the longevity of psychotherapy effects (Andrews et al. 2020; Lemmens et al. 2016; Singla et al. 2019; Vincent and Norton 2019; Zilcha‐Mano et al. 2018). If this interpretation were correct, the present data would indicate that results from previous LH studies may also have been influenced by sample characteristics, as the effect size would vary considerably across participants with the task acting as a trait‐activating context. Another interpretation of the present data is that the reactivation group underwent a similar intervention protocol as the reconsolidation group did. This idea is mainly based on comments by the participants following the physical training at T2; i.e., many participants reported that they were proud to have been able to complete the procedure despite having reached their physical limit long before the training ended. In fact, most participants could not complete the training as planned. Though all participants completed the full length of the training, most participants asked for a reduction of its intensity or not to increase intensity beyond a certain point. Based on this feedback, it is plausible that participants in the reactivation group experienced a similar procedure as the reconsolidation group (failing at the beginning before going on to succeed following their prolonged expenditure of effort), though the reconsolidation group experienced benefits of both cognitive and physical effort, while the reactivation group only experienced the positive effect of physical effort. Unfortunately, the present experimental design is not equipped to investigate the role of physical training, as it was planned only as a group‐independent facilitation of preceding effects (following the study by Keyan and Bryant (2017b); Keyan and Bryant (2017a)).

### 7.3 Inconsistent Results Regarding Midline Theta

Previous results (e.g., Soeter and Kindt 2015) indicated that reconsolidation‐based interventions may specifically affect implicit or somatic markers instead of explicit ratings. In this regard, the present study investigated midline theta as an electrophysiological correlate of motivation, control perception, and cognitive effort. According to the preregistered MLMM, we found that frontal midline theta (Fz) decreased in the reconsolidation group, while it increased in the reactivation group and did not change in the extinction group, following the results obtained from our pilot data. Along the same line, we found that more experience of failure in block 1 of T2 (indicating more reactivation of the experience of T1) was associated with larger decreases in theta activity across measurement occasions (T1–T3), providing evidence for the plausibility of the reactivation‐dependent mechanism behind group differences. Along this line, PFTA effects seemed mostly driven by frontal (Fz) influence, which is in line with the time frequency plots of Figure 6, showing that theta at Pz showed no particular theta response to the task.

Interestingly, this pattern stands in contrast to findings by Reznik et al. (2017), who reported that differences in PFTA effects were primarily explained by theta activity at Pz, which also distinguished between helpless and masterful experimental groups (also see Forster et al. (2023) for a similar finding). In light of this, the absence of a pronounced theta response at Pz in our study may be attributable to the fact that participants engaged in a helplessness task at both measurement occasions (T1 and T3). Although the anagrams presented at T3 were, on average, easier, the contrast between T1 and T3 may not be directly comparable to the distinction between helpless and masterful task conditions. Consequently, in the present study, PFTA effects were primarily driven by differences in theta activity at Fz, which may account for the divergence from previous findings.

Importantly, however, we argue that these results should be interpreted with caution. After reconsidering the preregistered modeling approach, we argue that the models underlying these estimates should have included an additional random slope for measurement occasion. Introducing this effect to preregistered models significantly improved model fit (indicating the validity of considering it in the analysis). Notably, though effect sizes did not change, all EEG‐related effects were rendered insignificant due to the large drop in degrees of freedom. Though the resulting model diverges from the registered approach, we argue that the present results should not be considered without the inclusion of this random effect. While the present results regarding EEG can serve as preliminary findings informing future investigations, the current study is probably not sufficiently powered for this (necessary) model complexity (note that we did not consider this random effect in the initial power simulation).

Interestingly, the effects for theta at Fz and PFTA could be rescued by adding BDNF levels after the intervention and training at T2 to the model. BDNF is related to learning and memory consolidation, with higher levels improving retrieval of (newly) learned information (see above). Notably, even when allowing a random slope for measurement occasion, we found that high BDNF levels increased theta only in the reconsolidation group, and relating thereto, in those who experienced more reactivation at T2. Though only 46 of the 60 total participants could be included in this analysis (due to missings in BDNF data), this effect remained stable even after exclusion of participants and trials that showed unproportional leverage on the model. Importantly, this result indicates that successful learning after reconsolidation was related to increases in theta at Fz, rather than decreases, as implied by the highly significant, albeit insufficient, modeling approach that was preregistered. Along these lines, our exploratory analyses implied beneficial effects of higher theta power at Fz, due to its correlation with higher anagram solving probability and motivation, which further solidifies the interpretation of unplausible results from the preregistered approach. Conversely, this exploratory analysis, including BDNF as a covariate, implied significant and plausible effects. Thus, though these results cannot be interpreted as confirmatory proof, the present manuscript highlights BDNF as a potential covariate of interest, conveying successful postretrieval extinction effects.

To further investigate whether large interindividual differences in the trajectory of EEG measures were the result of suboptimal preprocessing decisions, we further conducted a multiverse analysis considering 48 preprocessing pipelines (see Section 4.3 in the Supporting Information). Overall, though considerable variance in effects (sizes) was present, we found no indication that the preregistered approach was biased in any particular way.

## 8 Summary and Conclusions

In sum, the current study failed to provide confirmatory evidence for the superiority of reconsolidation‐based interventions in LH and, by extension, depression. Though the originally preregistered analyses indicated significant differences between the reconsolidation and other protocols on midline theta measures (PFTA and theta at Fz), we argue that these results should be interpreted with caution. As discussed above, the preregistered modeling approach was probably not complex enough for the data under investigation, requiring (nonregistered) adjustments that rendered initial findings insignificant. However, we were able to rescue the originally highly significant effects on theta by correcting for BDNF levels after the intervention, showing positive effects on theta for those individuals with higher BDNF levels, who experienced considerable reactivation before extinction training. Since higher levels of midfrontal theta were related to better task performance and self‐rated motivation, this finding may indicate that those individuals who experienced the reconsolidation protocol, showing physiological correlates of learning afterward, showed increased resilience at T3. Taken together, across self‐rated measures, performance ratings, and EEG parameters, the current data indicate substantial variability both inter‐ and intra‐individually, which seems to be far larger than the effects of interventions at T2. Though the experimental approach of this study worked largely as intended, this may indicate that the manipulation may have either been too weak to produce consistent effects across participants or that reconsolidation‐based interventions in the context of helplessness are strongly moderated by such interindividual differences. All in all, this study provided a first glimpse into a possible methodological approach to experimentally test the effect of reconsolidation‐based interventions in heterogeneous affective disorders such as depression. Given our results, future research should focus on the collection of larger samples, which may allow us to further discern the boundary conditions of the therapeutic method of reconsolidation‐based extinction learning.

## 9 Limitations

This study has several limitations that should be acknowledged. The most prominent is the relatively small sample size, which reduces both the robustness and the generalizability of our findings, particularly given the considerable interindividual variability in the data. Moreover, depression is a highly heterogeneous disorder, and our methodological approach was restricted to examining isolated facets (primarily motivation, effort, and attributional style) within a healthy sample rather than a clinical population. In addition, the experimental task relied on complex cognitive operations, which complicate the interpretation of EEG measures by making it difficult to attribute the observed effects to specific, well‐circumscribed cognitive functions. Finally, although we tested several EEG preprocessing pipelines, many alternative preprocessing choices remain unexplored. For example, in the present analysis, re‐referencing to the average was conducted before artifact correction; applying average re‐referencing after artifact correction might reduce the risk of artifacts being inadvertently spread during preprocessing.

## Author Contributions

**André Forster:** conceptualization, data curation, formal analysis, funding acquisition, investigation, methodology, visualization, writing – original draft, writing – review and editing. **Johannes Rodrigues:** formal analysis, visualization, writing – review and editing. **Billy Sperlich:** funding acquisition, project administration, resources, supervision, writing – review and editing. **Johannes Hewig:** funding acquisition, project administration, resources, supervision, writing – review and editing.

## Funding

This work was supported by the European Regional Development Fund, 1.2‐StMWKF.4‐UFR‐002 and Faculty of Human sciences, University of Würzburg, ID: 04 2022/3‐06.

## Conflicts of Interest

The authors declare no conflicts of interest.

## Supporting information


**Data S1:** psyp70217‐sup‐0001‐DataS1.docx.

## Data Availability

The data file used for the analyses, together with a detailed R‐script replicating all results, is available at https://doi.org/10.17605/OSF.IO/NT65F. This repository also contains the MATLAB code used for EEG preprocessing, exploratory multiverse analyses of the EEG data, and supplemental material outlining the advantages and disadvantages of methodological decisions. In addition, it provides further exploratory analyses, details from the pilot study, and an additional exploratory validation study of the materials. Due to the size and complexity of the various EEG preprocessing pipelines, individual EEG files have not been uploaded. However, these data can be made available upon reasonable request. Such requests should specify the preprocessing pipelines and steps of interest.

## Endnotes

^1^
A more detailed discussion of methodological decisions can be reviewed in Section 1 in the Supporting Information.

^2^
Please note that the idea of discrimination learning in the extinction group represents a post hoc explanation by participants to make sense of why they had very different experiences across test‐days. The task is not specifically built to induce discrimination learning, but this kind of idea may reflect the reasoning a participant may show to make sense of the situation.

^3^
Note that Reznik et al. (2017) found a positive relation between PFTA and motivation, while we found a negative one in our pilot data. Building on this, the direction of Hypothesis 2 diverges from what the literature may suggest.

^4^
In the pilot study, some participants tried to cheat by retyping the anagram into the non‐time‐restricted “solution screen,” giving them unlimited time to solve the trial. During data collection for this registered report, this action was addressed and prohibited by the experimenters.

^5^
In an additional exploratory analysis, theta at Pz and Fz will be analyzed for each electrode position individually.

^6^
As can be seen in the supplemental code, the model considering trial, satisfaction, and attribution could not be computed. To reduce model complexity and given, that inclusion satisfaction and attribution ratings may bias model results due to their positive relation with performance, their respective main effects were not considered when comparing mixed‐effects models as described in the hypothesis section.
