# Supplementary material for: The Use of Behavioral Reconsolidation Interference in Depressive Disorders. A Double‐Blinded Randomized Controlled Experimental Registered Report
Source: Psychophysiology. 2026 Jan 28;63(2):e70217. doi: 10.1111/psyp.70217 (PMC12851423; doi:10.1111/psyp.70217)
Supplement: Supplementary file 1 — Data S1: psyp70217‐sup‐0001‐DataS1.docx. [file PSYP-63-e70217-s001.docx]

This file contains supplemental information on the manuscript entitled: “The Use of Behavioral Reconsolidation Interference in Depressive Disorders. A double-blind randomized controlled experimental registered report” by Forster, Rodrigues, Sperlich & Hewig (2026), published in Psychophysiology

**Supplemental Information**

1. **Details and Considerations Regarding Experimental Design Decisions**
   1. **Arguments for and against the anagram task (T1, T3)**

The experimental task at T1 and T3 generally needed to fulfill the following requirements:

1. *The task should feature a trial-wise structure*. This improves the quality of subsequent EEG-based analyses and provides a straightforward way of interpreting data on the quantity of success/failure
2. *The task should feature trials with varying difficulty*. This provides data on a possible decline in performance (effort, motivation). If all trials were similar in difficulty, changes in ability cannot be assessed reliably (this can be seen in the details of item-response models in psychological diagnostics).
3. *The task should feature some kind of quantifiable feedback*. This allows feedback-related EEG analyses and follows the operationalization of helplessness as a reinforcement learning process, for which a prediction error can be scored in order to instill reactivation.
4. *The task should imply an internal, global, and stable attribute to be the main cause of failure*.
5. *From an ethical perspective, the task must not induce helplessness beliefs that may potentially lead to substantial issues in everyday life as participants cannot be debriefed after T1 or T2*
6. *The task must be largely unsolvable but also allow for trials that are potentially solvable*, as depression-related helplessness is defined as sustained passivity/anxiety despite being potentially able to (re)gain control
7. *The helplessness induced by the task must allow for some sort of quantifiable reactivation (T2) and repeated administration (T1 and T3)*

Along these lines, the classical helplessness literature offers few options. Though the classical unsolvable anagram task is well-established in the context of learned helplessness, it usually misses an inherent possibility to score maladaptiveness of passivity, as all anagrams are *per se* unsolvable. Instead, participants were often asked to solve a different (solvable) task after administration of unvolvable anagrams, which is not optimal in the present context, as we needed to ensure that all participants exited T1 with an overwhelmingly negative experience. If some participants were not helpless after the unsolvable anagrams, then they may have largely solved the consecutive second (solvable) task and thus produced variance in the experience taken from T1. As a result, we decided to modify the classical unsvolvable anagram task to include *per se* solvable trials. However, their difficulty was set so that it becomes unlikely that many trials are solved. This also has the advantage that from time to time, participants may solve a trial if they stay focused on the task, which allows them to score an interval-scaled performance score that is indicative of the participant's overall capability, combined with motivation/effort. Other helplessness-inducing tasks, like the one discussed in Reznik et al, (2017) or Forster et al. (2023), did not feature the required trial structure, distinct feedback, or quantification of motivation/effort through behavioral data. Finally, as the difficulty of items was in part manipulated by restricting the time participants had to solve them, we were also able to include a small number of trials that could generally be solved by participants as they lacked this restriction. As discussed in the main text, this allows for another way of continuously scaled quantification of motivation/effort without increasing the variance of experience at T1 too much.

In that sense, other stimulus material (e.g., arithmetic equations) could also be used for this task, as these may allow for the same general experimental design. In fact, in hindsight, mathematical equations may have been a better choice as the anagram task confounds eye movement with task-processing, which proved to be an issue in the analyses of EEG data. All in all, anagrams but also equations seemed a reasonable choice as participants may interpret their failure in the tasks as an indicator of their cognitive capacity, which may stimulate attribution to internal, global and stable traits that may not have a direct negative effect on everyday life (e.g., as compared to manipulating participants self-concept regarding social interactions/likability). Finally, both anagrams and equations generally allowed to present participants with the solutions to preceding trials, which should boost internal attribution.

Generally, the present data indicates that the task may have benefited from more trials, though participants frequently reported large exhaustion and depressed mood after 50 trials already (unfortunately, we did not record participants feedback to the experimenters systematically).

- 1. **Arguments for and against the arithmetic task (T2)**

Following the arguments of the previous section, the general procedure of T1 and T3 could also be conducted using equations instead of anagrams. When determining the task for T2, several additional requirements had to be met:

First, the task in T2 needed to differ from T1 in terms of location, stimulus material, and experimenters. The primary reason for this was to prevent unintended reactivation of the T1 experience. If the reactivation of helplessness occurred irrespective of the randomization in T2, the extinction group would also experience a subsequent prediction error, as for this group, T2 was designed to provide participants with a positive experience. This unintended reactivation could lead to uninterpretable results, as two groups may inadvertently undergo the same procedure.

On the other hand, presenting participants with a completely different task from T1 posed its own challenges. If the task differed too much, participants might not reactivate their T1 experience at all. This would create a similar issue, as there would be no meaningful difference between the reconsolidation and extinction groups. In this regard, we assumed that helplessness in depression is not necessarily tied to a specific trigger (following the classical interpretation of transsituationality) but rather to the experience of failure itself. We posit that passivity and anxiety arise as responses to the expectation of having no control over an outcome (e.g., winning vs. failing). This expectation should have been formed in T1 during the anagram task. Therefore, we argue that reactivating the helplessness-related conditioning process from T1 does not necessarily require the same anagram task but can also be triggered by a broader expectation of inefficacy.

Thus, a similar experience in T2, compared to T1, should be sufficient to reactivate the negative expectations initially formed around the anagram task. This process may be referred to as reactivation but could also be considered within the framework of generalization—two perspectives that are not mutually exclusive. Initially, participants may expect to perform differently in T2 when facing new stimuli or tasks. However, re-experiencing uncontrollable failure in this distinct situation may lead to a generalization of negative efficacy expectations. Based on these assumptions, we concluded that the benefits of avoiding unintended reactivation by using the same task and stimuli outweighed the challenges associated with using a different task or stimuli. Ultimately, we assumed that the expectation formed in T1 could be reactivated regardless of the specific task, as long as participants experienced a similar sense of helplessness. If this assumption held, then positive experiences following reactivation should lead to the reconsolidation of the original memory trace or, from another perspective, facilitate the generalization of positive experiences across both situations featuring the common expectation of helplessness.

Against this background, we adapted the *MIST* task specifically, as its underlying algorithm adapted the trial difficulty to a point where participants did not simply experience less failure but should have specifically experience that they still need to focus on the task to make it in time, binding effort/motivation to the outcome instead of just providing participants with easy wins (note that the task was reprogrammed in PsychoPy following the general idea of the MIST. We did not use the particular program by Dedovic, K., Renwick, R., Mahani, N. K., Engert, V., Lupien, S. J., & Pruessner, J. C. (2005)). In sum, this indicates that, if our assumptions regarding the reactivation of helplessness expectation independent from the task/stimuli at hand were wrong, the manipulation should not work, resulting in comparable experiences across the extinction and reconsolidation groups.

- 1. **Arguments for and against the ergometer training (T2)**

Based on a study by Keyan & Bryant (2017) we further decided to have all participants complete an ergometer training to boost consolidation and relatedly effect sizes. In hindsight, this decision may have potentially masked differences between the reactivation and reconsolidation groups. Though the reactivation group received no particularly positive experience in the arithmetic task, they may have felt a sense of positivity in the ergometer training. Generally, few participants were able to complete the ergometer training as intended. The majority of participants had to reduce the intensity at some point or at least not increase it as was required according to protocol. Still all participants trained for the preset amount of time, though finishing the training with lower intensities as was expected by the experimenters. It is plausible that participants thus learned that they could achieve much more than they expected if they just gave it their all. Though they may not have been able to complete the training as intended, they still may have had the experience that they could do more than they expected of themselves at the point of diverging from the protocol.

The present experimental design does not allow for the investigation of the ergometer training’s effect as all groups were tasked with completing the same procedure. We used this training to improve consolidation of the preceding (group-specific) experience, assuming that, if the training had no effect at all, this would not be an issue as the effect should be comparable across groups. As discussed above, this may have been a faulty assumption, given that the training itself could have had a similar effect as blocks 2-5 of the task may have had, which may have rendered the reactivation and reconsolidation groups similar by accident.

1. **Existing preliminary Data before the main study**

The study presented in the main manuscript was originally planned and started in 2019. Due to the Sars/Cov-II pandemic that emerged at that time, the study could not be carried out past the point of 16 participants. Of these data sets, many contained significant missings due to early technical issues that led to improper presentation and/or measurement of task features and responses. Nonetheless, the overall study design, including two test days with time-restricted anagram presentation on days one and three and the arithmetic task on day two, followed by a 20-min ergometer training, was carried out.

These existing data were not included in the final analyses but only used to explore the general validity of the experimental design and to provide estimates for variance-covariance matrices and effect sizes needed for subsequent sample size calculations. It should also be mentioned that some task properties were be changed with regard to the procedure as compared to those used to produce these pilot data (e.g., fixing the anagram’s lengths, see Section *Procedure*).

The following paragraphs show preliminary results of linear multilevel mixed models that were fit via the jamovi (Şahin and Aybek, 2019) module GAMLj (Gallucci, 2019). Results are only described briefly to give a first impression of this procedure’s validity.

- 1. **Pilot Data: Helpless Behavior**

Helpless behavior should be reflected by decreasing time spent on (solvable) anagrams before giving up. Furthermore, in the context of this study, this effect should show different trajectories across trials depending on the measurement occasion and the experimental group. The existing data shows no such three-way interaction in trials of wrongly solved anagrams without time restrictions (between measurement occasion, group, and trial). Instead, in a model comprising a random intercept for each participant (otherwise all effects are fixed) only a main effect for measurement occasion was present (*F*(1, 134.734)=6.427, *p* =.012), which indicates that the time participants spent on solving the anagrams before giving up, significantly decreased from T1 to T3. This is well in line with the idea of helplessness-related effortlessness. It thus indicates that participants built their behavior in T3 on the foundation of helplessness at T1. One reason for the missing effect concerning the three-way interaction may lie in the fact that in the presented version of the experiment, the anagrams differed substantially in their length, which complicates the estimation of within-day courses. Also, since the semantic knowledge about the helpless state at T1 may still exist, differences in behavior between the groups may not be present. Nonetheless, psychophysiological effects should be present otherwise.

- 1. **Pilot Data: EEG measures of Helplessness**

Following the results by Reznik et al. (2017) theta activity at electrode position, Pz minus Fz may be a suitable indicator for helplessness. Results from the existing data (all trials) show a significant interaction of group and measurement occasion, which is well in line with this idea (during the first 2 seconds of anagram presentation: *F*(2,1239.197)=215.353, *p*<.001) However, according to our pilot data, PFTA decreases in the reconsolidation group, while it increases in the extinction group, which contradicts the expected direction of effects found by Reznik and colleagues (see Figure 2). Substituting the grouping variable with the number of successful trials in block 1 of T2 (which is thought to indicate the amount of reactivation, inversely) shows a similar effect (*F*(1,395.115)=373.682, *p*<.001). More successful trials are linked to increased PFTA at T3. In this analysis, only the extinction and reconsolidation groups were included. These results further highlight the validity of the approach to use the number of successful (or failed) trials in block 1 of T2 as a metric predictor for the induction of potentially reconsolidation-related processes (see Figure 2). Interestingly, higher PFTA is predicted by less motivation (*F*(1, 1144)=30.078, *p*<.001), less satisfaction with one’s performance (*F*(1, 1042)=6.959, *p*=.008), and less internal attribution of failure (*F*(1, 1102)=42.923, *p*<.001) which indicates more favorable outcomes for the reconsolidation group in comparison to the control groups^[[1]](#footnote-1)^. Furthermore, motivation is positively correlated to greater RT (which adds to the idea of decreasing RT being related to helplessness; *F(1,120*)=13.0, *p*<.001)^[[2]](#footnote-2)^.


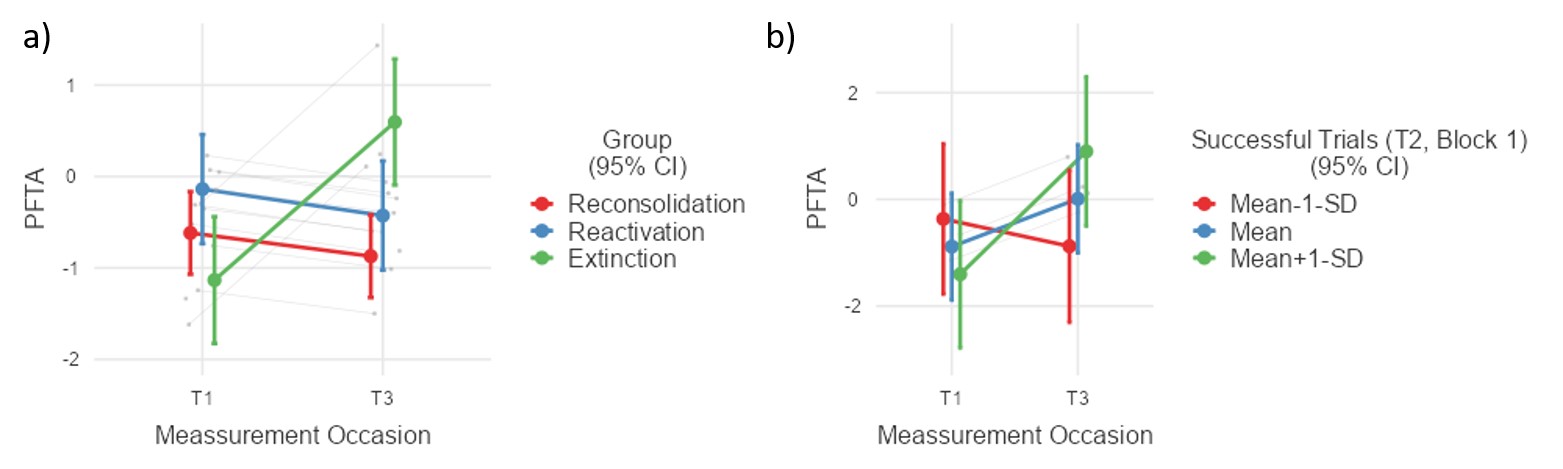


*a) Number of Observations: 1250, number of participants: 14. b) Number of Observations: 400, number of participants: 5. All Trials were used for this analysis. While the Extinction group increases in PFTA over the measurement occasions, the reconsolidation group decreases as a trend. This effect was replicated by including the number of trials that may reactivate the helplessness-memory: Participants that experienced greater success in block 1 of T2 (which should be most present in the extinction group) show increases in PFTA. Conversely, participants with more trials of reinstatement of failure, show decreases, which should especially be present in the reconsolidation and reactivation group. However, in b) the reactivation group was excluded.)*

1. **Additional Information on Analyses of the Main Manuscript**
   1. **Additional information on sample characteristics.**

The following table depicts the means and SDs of trait variables for each group:

| Group | Mean Age | N Women | BDI | Extraversion | Withdrawal | Industriousness | Volatility |
| --- | --- | --- | --- | --- | --- | --- | --- |
| Reconsolidation | 27.8 (11.6) | 11 | 4.55 (3.98) | 38 (5.68) | 22.6 (8.09) | 34.3 (9.44) | 24.3 (7.68) |
| Extinction | 25.3 (9.59) | 15 | 4 (4.18) | 37.6 (5.98) | 24.7 (6.73) | 33.55 (6.68) | 26.6 (4.99) |
| Reactivation | 25.4 (5.83) | 12 | 4.5 (4.19) | 38.3 (4.78) | 25.1 (5.89) | 31.85 (5.19) | 27.6 (5.80) |

- 1. **Probability of Failing (Behavioral Data, T1)**

All analyses described in this section can be reviewed in the section “Manipulation Check: Behavioral Data” within the supplemented R-code.

To estimate the linear trend in solving probability of time-restricted trials, two mixed-effects models were calculated and compared for model fit. In both models, a random intercept for each participant and a fixed effect for the trial were included. The trial-index was rescaled by dividing the trial index by 10 (preventing inflation of Eigenvalues, which may impair model estimation) and grand mean centered. In model two, a random slope for trial was further included in the analysis. According to the significant chi-squared test for model comparison, the inclusion of this random slope significantly improved model fit, which is why the results mentioned in the main manuscript were taken from this model.

Figure 4a indicated the absence of a linear decrease in performance to possibly result from the influence of an outlier (trial 42). As a result, this trial was excluded, and the random intercept/random slope models were refit. To further ensure that item difficulty was not unintentionally driving the resulting fixed effects, we decided to conduct an additional survey that was not preregistered beforehand. However, since the anagram order was fixed across all participants, trends in performance over time may generally be confounded with item difficulty. The survey was conducted online via the platform *SoSciSurvey*, presenting all anagrams in random order. Each trial consisted of the presentation of one anagram at a time. Once participants thought of the solution, they could proceed to the next screen, which asked for their answer and canceled the presentation of the anagram. Participants were instructed to solve the anagrams as fast as possible, though no time restriction was imposed. A total of 58 participants provided data for all anagrams. From this, item difficulty was calculated by computing the probability of participants solving an anagram within the time limits of the original study. This measure was then introduced to the random intercept/random slope model from before as a fixed grand mean centered covariate. As an alternative to this measure, we also computed the average of the time needed to solve any given anagram. The linear decrease in the probability to solve time-restricted anagrams was also robust after refitting this measure as a covariate (results can be reviewed in the supplemented R-Code, Section “Giving up in time-restricted anagrams).


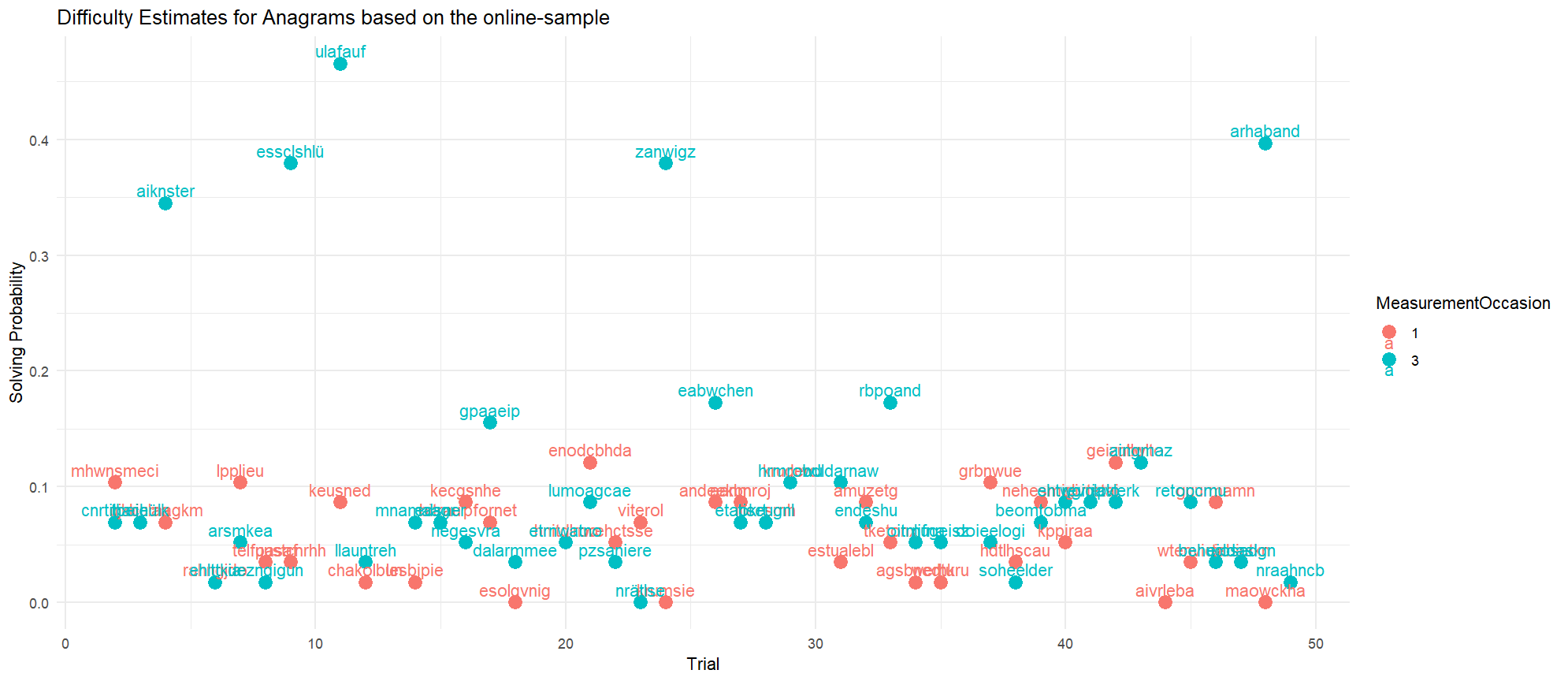
Our analyses show that items in measurement occasion T3 were on average easier than at T1 (p=.01). The following figure (T3= red, T1= blue) illustrates, that this is mainly the result of a few anagrams, while the majority of trials is similarly seldom solved across measurement occasions. The figure further shows that there was no indication of a generally increasing difficulty over time, reinforcing the interpretation of performance and motivation decreases in the lab-sample to be related to helplessness.

Another metric that may indicate changes in performance over time is the probability of ending time-restricted trials early (thereby failing to solve the anagram). We found no such trend (*p*>.5). However, only a total of 32 out of 2360 trials (60 participants, experiencing 40 time-restricted anagrams) were ended early by participants.

- 1. **Self-Rate Data (T1)**

Two mixed-effects models were fit for each screening-question (DV). Both models included *block* as a continuous, grand mean-centered fixed effect and *participant* as a random intercept variable. In model two, a random slope for *block* was added. Subsequently, the best model was evaluated following the same procedure as described before.

Regarding motivation, the inclusion of a random slope resulted in improved model fit, which is why this random intercept/ random slope model was used for further investigation. On average, motivation decreased over time, while no significant interaction between *block* (time) and *group* was found (Block: Extinction – Block:Reconsolidation: Estimate: -2.82, t(56)=-1.548, p=.127; Block:Reactivation – Block:Reconsolidation: Estimate: -1.02, t(56)=-.561, p=.576). However, since this type of analysis is not per se equipped to confirm the absence of an effect, this result should be interpreted with caution.

To further investigate whether this slope predicted performance in the first block of T2 (reactivation), which may negatively affect randomization, the reactivation was cluster-based centered for each group and used as DV. Notably, no such effect was found (p>.4), increasing confidence in the randomization process. Details for these analyses can be found in the R-Code, Section *Reactivation and Personality*.

1. **Additional (Exploratory) Analyses**
   1. **Relation between performance, motivation, and midfrontal Theta.**

In an additional analysis, we found that motivation ratings of a given block significantly predicted the probability of solving an anagram (*z*=3.583, *p*<.001). Regarding The relationship between midfrontal theta and performance, we found that theta at Fz was only then predictive of being able to solve an anagram (*z*=3.332, *p*<.001), when alpha power at the same electrode was also considered (which was negatively related to anagram solving: *z*=-3.789, *p*<.001). Notably, alpha power at Fz was positively related to ratings concerning internal attribution (*t*(50.138)=-2.687, *p*=.010; note that smaller values indicate more internal attribution), while theta was positively related to motivation (*t*(34.808)=2.118, *p*=.041), indicating functional differences between alpha and theta at Fz.

- 1. **Reactivation at T2 as Dependent Variable.**

To check whether group- or interindividual differences across measurement occasions were the result of preexisting traits, the number of correctly answered trials in block 1 of T2 (indicating the reactivation of helplessness from T1) was used as a dependent variable, while trait estimates and measures obtained from T1 were the IVs. If both the reactivation at T2 and the change of EEG activity from T1 to T2 were predicted by these variables, then this would indicate that potential reconsolidation-based effects may rather constitute trait-like resilience effects or at least be trait-mediated. As can be seen from the supplemented R-Code (Section Reactivation and Personality), we did not find any questionnaire- or self-rate measure obtained at T1 to predict the number of failed trials in block 1 of T2.

- 1. **Exploratory Multiverse Analysis.**

Though the preprocessing pipeline for the main manuscript was preregistered, we observed suboptimal performance of this approach, given some specific issues in the data. Ultimately, the preprocessing pipeline producing the data of the main manuscript was carried out following the preregistration except for the addition of applying line noise filters. However, to also provide some insight into the impact of this possibly suboptimal preprocessing, we additionally report the results of an exploratory multiverse approach to preprocessing below. The decision on which preprocessing approaches to evaluate was made against the background of following issues:

a) substantial influence of line noise at 50Hz, which is supposedly the result of a technical dysfunction in the amplifier leading to increased power of artifacts from the electrical power grid. To address this, we included Notch filters for 50Hz and its harmonics before channel rejection. Not doing so resulted in considerable issues during IC decomposition as many ICs picked up on the line noise, allocating so many ICs toward this issue that few ICs remained to pick up on other artifacts. This in turn led to ICs conflating noise with real data.

b) Due to the long time it took to complete data acquisition, many experimenters had to be trained to collect the data, leading to significant fluctuations in data quality. This in turn increased the need to adapt the preprocessing pipeline in terms of both trial and IC rejection policies.

c) Supposedly due to varying IC decompositions for T1 vs. T3, it could not be ruled out that the effect of interest could have been partly purged from the data in one measurement occasion but not the other, which may lead to large interindividual variance in the trajectory between measurement occasions. To circumvent this, we tested the results of ICAs that were trained on the data from both measurement occasions vs. ICAs that focused on each data set individually.

d) Due to the correlation between eye movement (supposedly to assess and process the anagrams) with cognitive processing, we found that the classical *runica* algorithm sometimes provided suboptimal solutions. We thus investigated whether another algorithm (*sobi*) would improve the results.

e) Finally, to rule out that measures across measurement occasions were just the result of slight differences in the location of the effect (e.g., following systematic issues in cap placement across experimenters), we computed not only the preregistered CSD reference, but also an average reference, which supposedly does not rely as much on slight variations of the effect across electrodes. On the other hand, this approach is likely to lose spatial specificity of effects, which may impair the comparison (indexing) between signals from Fz and Pz, as volume conduction will make them more similar.

f) Finally, we varied the number of cycles used for wavelet convolution (3.5 as preregistered vs. 30), investigating differences in results building on the tradeoff between temporal and spectral precision.

**Main Takeaways**

1. Theta activity at electrode position Pz may not be particularly meaningful. Instead, the main response is within the alpha band
2. The theta response at Fz seems to contain two distinct components: an early one (0-500) and a late one (500-2000+)
3. The extinction group shows a prominent alpha response at Fz at T1, which may distort results if the alpha response fulfills the same function as the theta response in other groups or individuals
4. Topographies indicate that theta effects at Fcz are more pronounced than at Fz
5. Joint IC decomposition led to more homogenous results across groups, diminishing effects of measurement occasion
6. Mara often led to larger effect sizes than ICLabel

Following these considerations, many more analyses were conducted, which are not discussed here due to the large number of necessary figures, information, and tables. We refer the reader to the supplemented R-Code to compute their comparison of interest.

- 1. **Multilevel modeling approach in hypotheses 1-3**

As noted in the main manuscript, we decided to add a random slope that was not part of the registered analysis plan to the models. We did so because the inclusion of this model term led to considerable increases in model fit. From a theoretical point of view, consideration of measurement occasion as a random slope ensures that the model does not “borrow” its degrees of freedom for estimates on measurement occasion from observations on level 1 (individual trials). As a result, the degrees of freedom drop dramatically, since the model now only has 2 (measurement occasions) * 60 (participants) degrees of freedom, instead of 50 (trials) * 2 (measurement occasions) * 60 (participants). Following this, the variance in changes across measurement occasions that is not the result of (fixed) group effects is now caught in the random slope term. Therefore, model fit increases proportionally to the variance that can now be explained by this term (the random slope). Not including this term (in accordance with the preregistration) assumes that all differences across measurement occasions were either the result of (fixed) group differences or (fixed) general differences across measurement occasions (which would be equal for all individuals). Since the random slope for measurement occasion had such an impact on the model, our results indicate that large interindividual differences in theta activity across measurement occasions were present; trajectories across measurement occasions that were independent from the group. Furthermore, our analyses showed no indication that this random variance could be explained by including reactivation at T2 as a covariate. This indicates that interindividual differences across measurement occasions were not, to a large extent, the result of interindividual differences in reactivation, reinforcing the idea that this variability is the result of preexisting participant traits rather than a byproduct of interindividual differences in the experience with the task/manipulation. All in all, this alludes to the idea that changes in the EEG, but also performance ratings (see hypothesis 1) may be subject to preexisting interindividual traits rather than experimental manipulations. Still, there are trends toward group differences to be found even after inclusion of the random slope, but the present data may not have the appropriate power to reliably test for them.

- 1. **Including BDNF as a covariate.**

Including mean BDNF levels after the intervention at T2 in the analyses of Hypothesis 2 rescues the original findings that vanished after allowing a random slope for measurement occasion. Notably, only 46 participants could be included in these analyses due to missings in BDNF assessment. All results can be reviewed in detail using the supplemental R Code and dataset. In brief, allowing a three-way interaction of BDNF, measurement occasion, and group/reactivation showed that theta at Fz increased for those participants with (relatively) increased BDNF levels while it decreased for those with relative low levels. Such effects were not present in the other groups (measurement occasion * extinction-reconsolidation * BDNF: t(40)=-3.78, p<.001; measurement occasion * reactivation-reconsolidation * BDNF: t(40)=-3.892, p<.001; measurement occasion* reactivation at T2, * BDNF: t(32)=-3.305, p=.002). Similar results were found concerning PFTA (measurement occasion * extinction-reconsolidation * BDNF: t(40)=2.909, p=.006; measurement occasion * reactivation-reconsolidation * BDNF: t(40)=2.707, p=.010; measurement occasion* reactivation at T2, * BDNF: t(32)=1.804, p=.081), as can be reviewed in Figures 8G and 8H. Even after excluding participants with unplausible leverage on the data (n=3), these effects remained significant, as can be seen in the R-code. The effect of BDNF are illustrated below.


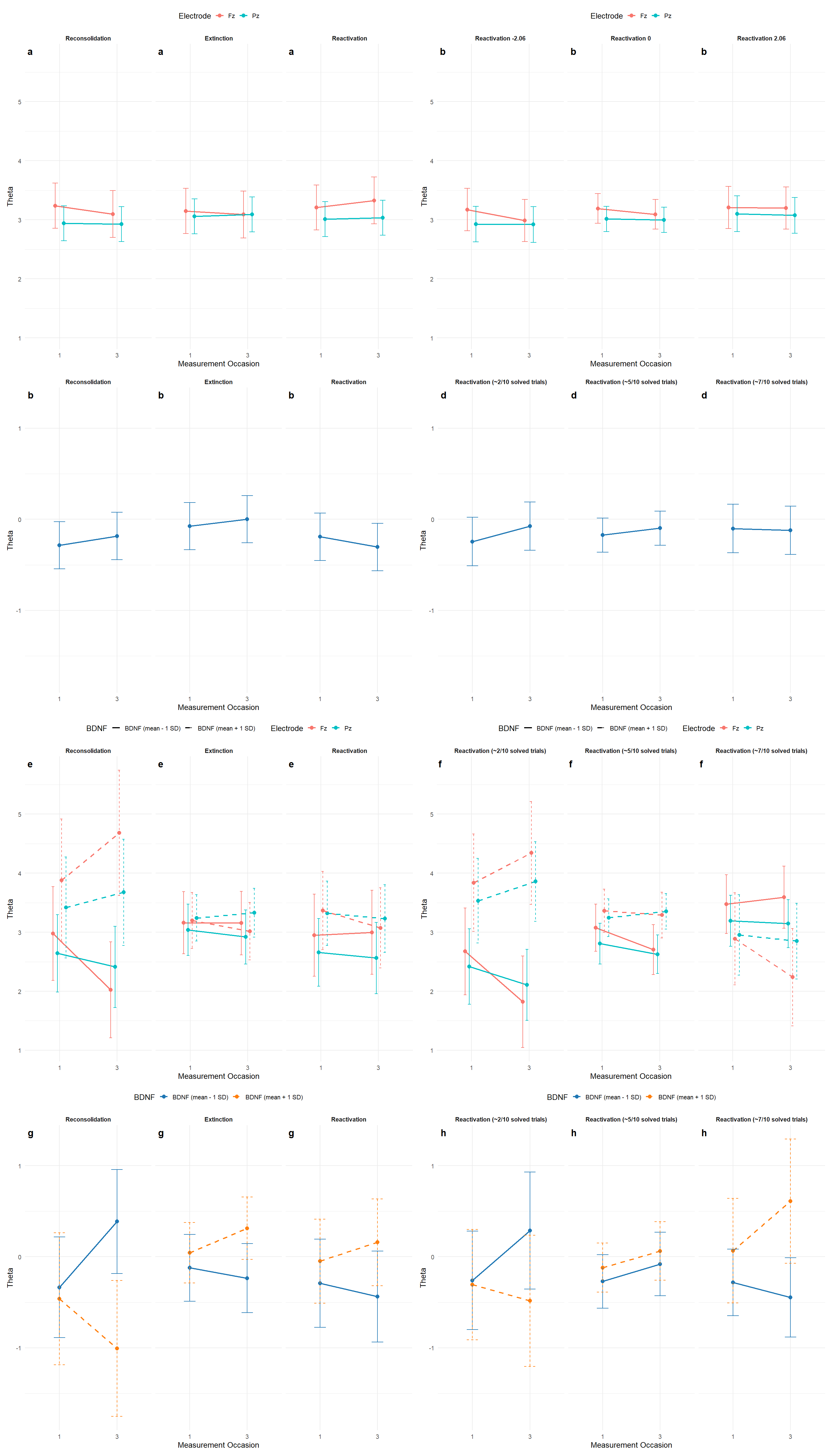


1. Additional Information

*Illustration of the EEG setup*

1. These results follow from a mixed model approach with PFTA as dependent variable, motivation, internal attribution, satisfaction with one’s performance and the feeling of becoming better in the task as fixed effects. A random intercept was included for each participant. Only main effects were allowed [↑](#footnote-ref-1)
2. This participant-based random intercept model included reaction time as dependent and motivation as cluster-centered independent variables. [↑](#footnote-ref-2)
